# Unraveling the Bivalent and Rapid Interactions Between a Multivalent RNA Recognition Motif and RNA: A Kinetic Approach

**DOI:** 10.1021/acs.biochem.4c00301

**Published:** 2024-10-14

**Authors:** Guillermo Pérez-Ropero, Anna Pérez-Ràfols, Tommasso Martelli, U. Helena Danielson, Jos Buijs

**Affiliations:** † Department of Chemistry − BMC, Uppsala University, Uppsala SE 751 23, Sweden; ‡ Ridgeview Instruments AB, Uppsala SE 752 37, Sweden; § Department of Chemistry “Ugo Schiff″, Magnetic Resonance Center (CERM), University of Florence, Florence 50019, Italy; ∥ Giotto Biotech s.r.l, Sesto Fiorentino, Florence 50019, Italy; ⊥ MRC Protein Phosphorylation and Ubiquitylation Unit, University of Dundee, Dundee, Scotland DD1 5EH, U.K.; # Science for Life Laboratory, Drug Discovery & Development Platform, Uppsala University, Uppsala SE 751 23, Sweden; ¶ Department of Immunology, Genetics and Pathology, Uppsala University, Uppsala SE 751 85, Sweden

## Abstract

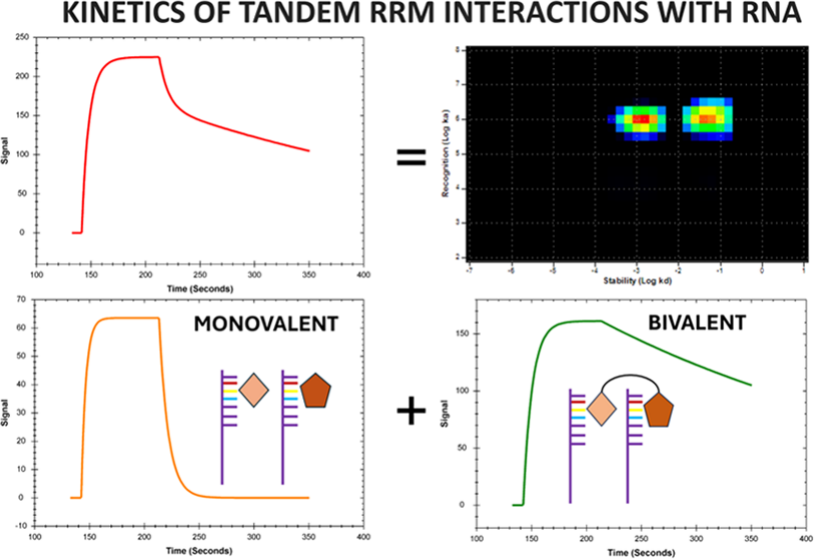

The kinetics of the interaction between Musashi-1 (MSI1) and RNA have been characterized using surface plasmon resonance biosensor analysis. Truncated variants of human MSI1 encompassing the two homologous RNA recognition motifs (RRM1 and RRM2) in tandem (aa 1–200), and the two RRMs in isolation (aa 1–103 and aa 104–200, respectively) were produced. The proteins were injected over sensor surfaces with immobilized RNA, varying in sequence and length, and with one or two RRM binding motifs. The interactions of the individual RRMs with all RNA variants were well described by a 1:1 interaction model. The interaction between the MSI1 variant encompassing both RRM motifs was bivalent and rapid for all RNA variants. Due to difficulties in fitting this complex data using standard procedures, we devised a new method to quantify the interactions. It revealed that two RRMs in tandem resulted in a significantly longer residence time than a single RRM. It also showed that RNA with double UAG binding motifs and potential hairpin structures forms less stable bivalent complexes with MSI1 than the single UAG motif containing linear RNA. Substituting the UAG binding motif with a CAG sequence resulted in a reduction of the affinity of the individual RRMs, but for MSI1, this reduction was strongly enhanced, demonstrating the importance of bivalency for specificity. This study has provided new insights into the interaction between MSI1 and RNA and an understanding of how individual domains contribute to the overall interaction. It provides an explanation for why many RNA-binding proteins contain dual RRMs.

## Introduction

Most biological processes are mediated by proteins that interact with other biomolecules, often forming complex networks that are regulated at different levels.^1^ Since the central dogma of molecular biology was established and the role of nucleic acids as coding information for protein expression became clearer,^2^ focus shifted toward DNA transcription and RNA translation as the key processes that regulate protein expression, thereby defining the overall cellular homeostasis.^3^

RNA-binding proteins (RBPs) are among the most abundant proteins in the cell. They interact directly with RNA and thereby regulate splicing, expression, localization, and stability.^4,5^ There are several families of RBPs depending on the structural characteristics of their domains, the most common being the RNA Recognition Motif (RRM).^6^ RRM-containing proteins harbor one or several domains with a characteristic αβ sandwich structure and a βαββαβ distribution that enables binding to single-stranded RNA.^7^ RRMs are found in various organisms, including prokaryotes and eukaryotes, and can even be found in viruses.^8^ They are involved in a wide range of cellular functions, such as proliferation and differentiation.^9^ RBPs, and RRMs particularly, are involved in the formation of phase-separation structures, including membraneless cellular compartments that have an important role in regulating translation.^10,11^ Multivalent interactions are a key factor for these compartments to appear and thereby modulate the biological activity through establishing effective concentrations that profoundly affect the bound/unbound ratio.^12^ Recently, advances in RNA therapeutics have strongly enhanced the interest in approaches to use RNA-RBP interactions to affect the expression of until now undruggable targets.^13^

In the present study, we explored the kinetic characteristics of the interaction between Musashi-1 (MSI1) and RNA. Human MSI1 is a 39 kDa RRM protein with 362 amino acids that contains two *N*-terminal RNA-binding domains, RRM1 and RRM2. It was originally identified in drosophila, where it was found to be involved in sensory organ development.^14^ In mammalian cells, MSI1 was first discovered in mice and initially is connected to neural cell differentiation and development.^15^ It has later been found to play a role in several different human pathologies, including cancer^16^ and neurological diseases.^17^ Several pathways, including Notch and Wnt, are regulated through the MSI1-RNA interactions. For Notch, MSI1 binding to Numb mRNA reduces translation and thereby upregulates the pathway favoring differentiation and proliferation.^18^ In addition to the two RRMs, MSI1 contains a long *C*-terminal intrinsic disorder region (IDR). This region has been associated with protein aggregation^19^ and the formation of cellular condensates, such as stress granules in glioblastoma, which have been correlated with a worse prognosis.^20^

MSI1 was initially reported to specifically bind to a (G/A)U_1–3_AGU sequence motif,^21^ but the essential core sequence has later been reduced to the three-nucleotide UAG sequence.^22^ Various aspects of the MS1-RNA interaction have been studied, but few details are known about the involvement of the individual RRM1 and RRM2 domains in the overall interaction mechanism and the significance of embedding two RRMs that target similar UAG segments in the protein. It has been proposed that the binding is multivalent and that RRM1 is responsible for the sequence recognition while binding at RRM2 increases the affinity, although there is no experimental evidence for the role of RRM2.^22,23^

To this day, there are no extensive studies that provide experimental information on the kinetics, mechanism, or affinity of MSI1-RNA interactions for the individual RRM1 and RRM2 domains or the two in combination (MSI1 1–200) in a single study. Mechanistic and kinetic information are the keys to understanding the multivalent interaction process, how it influences the biological function of MSI1, and how to design compounds with a potential therapeutic effect.^24^

To decipher the characteristics of the interactions of the two RRMs and the mechanism of the bivalent interactions between MSI1 and RNA, we used a surface plasmon resonance (SPR) biosensor. SPR-based biosensors are extensively used in the characterization of biomolecular interactions, as they enable the study of interactions in real-time with high sensitivity and flexible assay design.

A new method was developed to quantify the affinities for the fast bivalent interactions observed. This method was used to investigate the impact of variations in the RNA sequences. It is expected that this method will be relevant not only for the understanding of the MSI1 function but also for other rapid and bivalent interactions.

## Materials and Methods

### Cloning, Expression, and Purification of RRM1 and RRM2 Domains of Human MSI1

To produce RRM1 of human MSI1 (aa 1–103), a pET29b plasmid (Twist Bioscience HQ, CA) with an *N*-terminal Strep-tag followed by the DNA coding for the first 261 residues of human MSI1 (Uniprot code: O43347) was modified by site-directed mutagenesis to replace Met-104 with a stop codon. To produce RRM2, the DNA coding for residues 104–200 of human MSI1 followed by a *C*-terminal Strep-tag was cloned in a pET21a plasmid between NdeI and XhoI (GeneScript, UK).

The recombinant plasmids containing the DNA sequences coding for RRM1 and RRM2 were overexpressed in *E. coli* BL21(DE3) cells. Cells were grown in Luria Broth at 37 °C until the optical density at 600 nm (OD600) reached 0.6–0.8. Subsequently, expression was induced with 0.5 mM isopropyl β-D-thiogalactoside (IPTG). Cells were incubated at 37 °C for 3 h and then harvested by centrifugation at 4 °C, for 15 min at 7500 rpm. The cell pellet was resuspended in lysis buffer (100 mM Tris-HCl pH 8.0, 150 mM NaCl, 1 mM EDTA, and SIGMAFAST Protease Inhibitor Cocktail tablets (Sigma-Aldrich, Milan, IT)), ruptured by sonication, and separated by centrifugation at 30,000 rpm for 35 min at 4 °C. The soluble fraction was filtered with a 0.22 μm membrane and purified using a Strep-Tag HP 5 mL column. The eluted fractions containing protein were collected and purified to homogeneity by size exclusion chromatography using a Hi load 26/60 Superdex 75 pg column, equilibrated with 50 mM Tris-HCl pH 7.5, 150 mM NaCl, 0.5 mM EDTA, and Protease Inhibitor Cocktail. Fractions in the peak containing protein of interest were collected and treated with a 5% polyethylenimine (PEI) solution to remove any DNA/RNA attached to the protein (procedure described below). PEI (Sigma-Aldrich, Milan, IT) as a 5% (w/v) solution, pH 7.9, was added slowly under stirring to the cell extract, and the resulting suspension was centrifuged at 7500 rpm for 30 min. The amount of PEI used for the removal of nucleic acid was estimated by using the following equation: (*V*_cell extract_ + *x*) × 0.8 = PEI % * x, where *x* is the amount of PEI in mL.

The pellet was discarded, while the supernatant was treated with solid ammonium sulfate, which was added slowly under stirring at 4 °C until reaching 70% (w/v) to eliminate excess PEI in solution. The precipitate, containing the protein, was collected by centrifugation and washed (4–5 times) with 30–60 mL of a 70% (w/v) ammonium sulfate solution. After each washing step, the precipitate containing the protein was collected by centrifugation and resuspended in 70% (w/v) ammonium sulfate solution. After washing, protein precipitate was resuspended in the desired buffer. RRM1 was resuspended in RNase-free final buffer 1 (50 mM Tris-HCl, pH 7.5, 140 mM NaCl, 0.5 mM EDTA, and Protease Inhibitor Cocktail) and filtered with a 0.22 μm membrane, whereas RRM2 was resuspended in RNase-free final buffer 2 (50 mM Tris-HCl, pH 7.2, 140 mM NaCl, 1 mM EDTA, and Protease Inhibitor Cocktail). Successful removal of the nucleic acid was confirmed by UV–vis. The maximum of the UV absorption was shifted from 260 nm (RNA/DNA bound) back to 280 nm (RNA/DNA free).

### Cloning, Expression, and Purification of Human Musashi-1 (MSI1) RRM1-RRM2 Tandem Domain

MSI1 is here a truncated version of human MSI1, encompassing the RRM1 and RRM2-containing *N*-terminal regions of recombinant human MSI1 (amino acids 1–200). The gene encoding this region was inserted into plasmid pET29b and overexpressed in BL21(DE3) GOLD *E. coli* cells. The protein was produced and purified, as described previously.^25^

## RNA

RNA strands with a 3′-biotin group were obtained from Metabion, Planegg, Germany. RNA L1 contains a single UAG motif, while RNA-L1a contains a CAG motif instead. RNA-HP2a binding to MSI1 has previously been described,^21^ and RNA-HP2b was designed based on the doublecortin (DCX) mRNA, a MSI1 endogenous ligand.^26^ The potential for RNA HP2a and HP2b for forming hairpins was analyzed using the RNAComposer software.^27^

### SPR Biosensor Analysis

SPR biosensor experiments were performed using Biacore 3000 and Biacore T200 instruments (Cytiva, Uppsala, Sweden). For immobilization, the RNAs were diluted in running buffer (50 mM Tris, 140 mM NaCl, 0.5 mM EDTA, and 0.05% Tween 20) to a 100 nM concentration. The RNA solution was subsequently injected over a streptavidin-coated sensor chip (SCB-SAD 200 M and SCBS-SAD 200M, Xantec, Dusseldorf, Germany) at a flow rate of 2 μL/min until the desired Response Unit (RU) level was achieved. Experiments were performed with various immobilization densities ranging from 39 to 1390 RUs.

MSI1, RRM1, and RRM2 were diluted 2-fold in running buffer to create a concentration series ranging from 1.9 nM to 1 μM. The surface was regenerated by injecting a 1 M NaCl and 1 M MgCl_2_ solution for 60 s. All experiments were performed at 25 °C. MSI1 experiments were performed with a 100 μL/min flow rate, and RRM1 and RRM2 experiments were performed with a flow rate of 60 μL/min. Association times ranged from 1 to 2 min and dissociation from 2 to 3 min.

### Interaction Kinetic Models

**Scheme 1:**
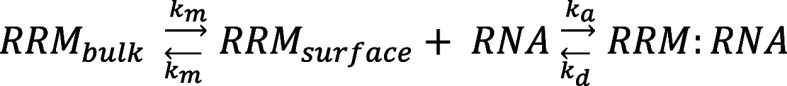


The 1:1 model accounts for the reversible interaction between an analyte and an immobilized binding partner with the formation of a single complex in one step, being the simplest interaction model. For rapid interactions, the model is extended by an additional first step represented by the mass transfer coefficient (*k*_m_) to account for an initial binding rate that is limited by the mass transport of analytes in solution to the immobilized binding partner on the sensor surface (Scheme 1). The scheme assumes a single RRM interacting with RNA, but the 1:1 model (without accounting for the mass transport step) was also used as the first option for evaluating interaction curves for MSI1.

The affinity is defined by the equilibrium dissociation constant (*K*_D_), i.e., the ratio between the concentrations of free and bound molecules, which is also equal to the ratio between the dissociation and association rate constants (*k*_d_ and *k*_a_, respectively) (eq 1).
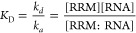
1

**Scheme 2:**
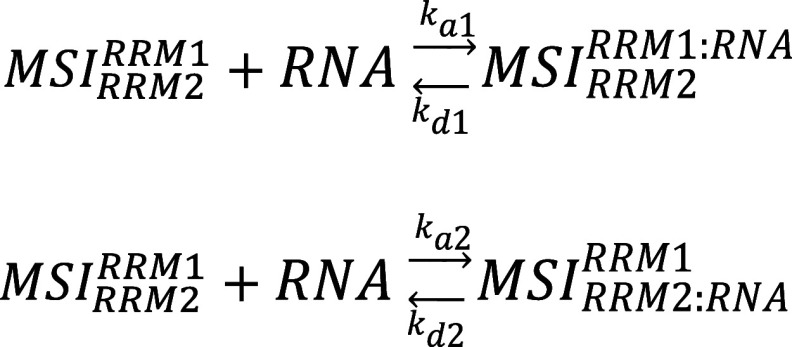


The heterogeneous ligand model (1:2) accounts for two independent 1:1 interactions, generating two different complexes (Scheme 2). This model could, for example, describe the interaction between MSI1 and an immobilized RNA containing one UAG sequence.

The *K*_D_ values of each of the two interactions (*K*_D1_ and *K*_D2_) are obtained as shown in eq 1.

**Scheme 3:**
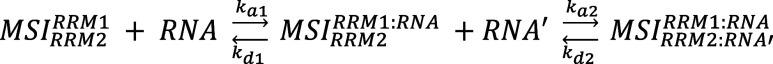


The bivalent analyte model (2:1) can potentially describe a 2-step interaction of MSI1 with two oligonucleotides simultaneously resulting in a bivalent complex, as shown in Scheme 3. (Here illustrated for the case of RRM1 binding first, but the order of binding is not defined).

The two dissociation constants (*K*_D1_ and *K*_D2_) are defined by eqs 2 and 3.
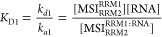
2
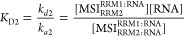
3

The second interaction does affect the shape of the curve, but the interaction itself does not result in a signal change.^28^ Note that the second interaction step in Scheme 3 is dependent on the concentrations of bound MSI1, and the concentration of free RNA on the sensor chip rather than on the unbound MSI1 concentration in solution. Evaluation software expresses the surface concentrations in signal units and the actual surface concentration expressed in Molar can be estimated from the signal as it is known that 1 RU corresponds to roughly 1 pg/mm^2^ of molecules on the sensor chip,^29^ and the thickness of the hydrophilic dextran polymer layer onto which molecules are immobilized is roughly 200 nm (according to the manufacturer). Thus, for MSI1 with a molecular weight of 22.6 kDa, a binding signal of 1 RU corresponds to a concentration of approximately 0.22 μM.

**Scheme 4:**
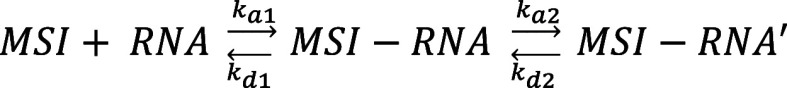


The induced fit model describes the process when a complex is formed in a first step followed by a rearrangement into a stable complex in a second step from which it cannot directly dissociate unless it goes back to the less stable complex form, as illustrated in Scheme 4.

### Data Analysis and Parameter Estimation

Before analysis, signals were double reference subtracted, first by subtracting signals from RNA-coated surfaces from an RNA-free reference surface, and second by subtracting signals from buffer injections from signals of MSI1 and RRM-containing sample injections.

For each experiment, four to ten consecutive concentrations containing the most informative data^30^ were selected for nonlinear regression analysis using the biophysics version of TraceDrawer 1.9.2 and 1.10 (Ridgeview Instruments AB, Uppsala, Sweden). To verify the performance of the fitting process, some experimental data were evaluated using BiaEval and Biacore T200 evaluation software (Cytiva, Uppsala, Sweden).

The goodness of fit and the values of the parameters defined by the interaction models were estimated using global nonlinear regression of complete sensorgrams for the series of analyte concentrations injected. Statistical information on the fitting accuracy was obtained by comparing χ^2^-values relative to the highest signal obtained at the highest concentration employed. The robustness of the fitting process was evaluated by comparing the results obtained by using different starting values for the fitted parameters. The reproducibility was assessed by the variation in estimated values between the replicates. RRM1 and RRM2 binding to RNA L1 and L1a, RRM1 binding to HP2a and HP2b, and MSI1 binding to RNA L1a, HP2a, and HP2b were performed in triplicates. RRM2 binding to RNA HP2a and HP2b were performed in quadruplicates, and MSI1 binding to RNA L1 included seven replicates. Average values for the association and dissociation rate constants and *K*_D_-values are presented together with the coefficient of variation (CV %), i.e., the ratio of the standard deviation to the mean. All fitting models had *B*_max_, which represents the signal when all immobilized molecules are bound by analytes, fitted individually for each curve to account for the variation of the number of targets (RNA) after each concentration and regeneration cycle that was able to bind RRMs. As no bulk signal was observed in the double reference subtracted sensorgrams and to improve robustness in the fitting process by avoiding that rapid association and dissociation events were fitted as bulk signal, the bulk index was set as a constant with a zero value. For MSI1-RNA interactions that were analyzed with more complex models (Schemes 2–4), starting values for *k*_a1_ and *k*_d1_ were set to values close to those observed for the RRM-RNA interactions and amounted to 1 × 10^6^ M^–1^·s^–1^ and 3 × 10^–1^ s^–1^, for *k*_a_ and *k*_d_, respectively. For all other parameters, default starting estimates were used. Potential cooperativity between binding of the two RRM domains to RNA was evaluated through determining the Hill coefficient^31−33^

Experimental data were also analyzed using InteractionMap (Ridgeview Diagnostics AB, Uppsala, Sweden). This software estimates the presence and rate constants of multiple 1:1 interactions and their contribution to the overall binding process.^34^ Mathematically, InteractionMap applies an iterative process to search for potential independent 1:1 interactions and estimates how much each interaction contributes to the overall measured binding curves. Visually, individual interactions are presented in *k*_*a*_ – *k*_*d*_ plots with heat map colors to indicate the relative contribution of each interaction peak.

## Results

### RNA Biosensor Surfaces for MSI1 Interaction Studies

Five different RNAs were used to generate biosensor surfaces for MSI1 interaction analyses (Table 1). Four were based on the characteristic MSI1 binding motif, (G/A)U_*n*_AGU. Of these, two contained a single binding motif (RNA L1 and RNA L1a) and differed in the first position of the UAG binding motif (U vs C), while two encompassed two copies of the central binding motif (RNA HP2a and HP2b). The latter were selected based on the sequence of natural binders DCX and Numb. Both have been described as forming hairpin-like structures, which was supported by computational modeling of their structures. A nine nucleotide RNA that did not contain the MSI1 binding motif was used as a negative control. Neither the RRMs nor MSI1 interacted with the negative control surface (Figure S1). The effect of different surface densities on the interaction with MSI1 was evaluated for RNA-L1.

**Table 1:** RNAs Used for the Generation of SPR Biosensor Surfaces^a^

name	feature	sequence
RNA-L1	single motif	5′-uug uua guu acc ccu u-3′
RNA-L1a	single motif	5′-uug uca. guu acc ccu u-3′
RNA-HP2a	dual motif	5′- aag cgu uag uua uuu agu cgc uu −3′
RNA-HP2b	dual motif	5′- cac ucu gua gua ugu agg guu uau uu −3′
RNA C	negative control	5′-cgg cgc cgc-3′

a All were 3′-biotinylated.

### RRM1 and RRM2 Interactions with RNA

**Figure 1:**
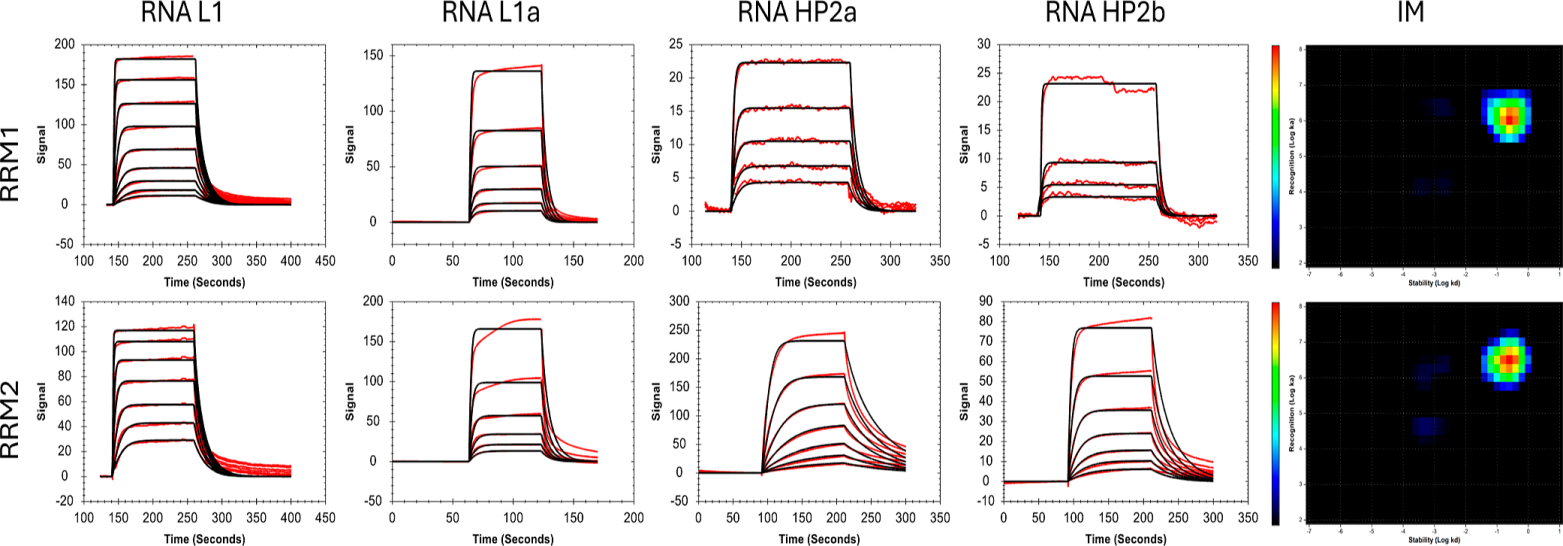
Representative sensorgrams (red) for RRM1 and RRM2 interactions with four different RNA strands. Sensorgrams from regression analysis using the 1:1 model (Scheme 1) are shown in black. The concentration ranges for the interaction pairs were: RRM1-RNA-L1 1.9 to 500 nM; RRM2-RNA-L1 7.8 to 500 nM; RRM1 and RRM2 to RNA-L1a 15.65 to 500 nM; RRM1-RNA-HP2a 3.9 to 62.5 nM; RRM1-RNA-HP2b 7.8, 15.65, 31.25, and 62.5 nM; and RRM2 to RNA-HP2a and HP2b 1.9 to 125 nM. The interaction maps (IM) for RRM1-RNA-L1 and RRM2-RNA-L1 interactions are shown.

Both RRM1 and RRM2 showed rapid interactions with all binding motif containing RNAs. A 1:1 model that takes mass transport into account (Scheme 1) fitted well to the data (Figures 1 and S2–S9). The estimated association and dissociation rate constants, and the *K*_D_-values are summarized in Table 2. The single spots in the interaction maps for RNA-L1 also show that the RRM1 and RRM2 interactions were well described by a 1:1 interaction model (Figure 1, right panels).

**Table 2:** Summary of Kinetic Parameters for RRM Interactions with Various RNAs, as Estimated by Regression Analysis Using the 1:1 Model^a^

RNA strand	RRM1			RRM2		
	*k*_a_ (M^–1^ s^–1^)	*k*_d_ (s^–1^)	*K*_D_ (nM)	*k*_a_ (M^–1^ s^–1^)	*k*_d_ (s^–1^)	*K*_D_ (nM)
RNA-L1	3.5 × 10^6^ (8.2)	0.17 (11)	47 (2.6)	4.6 × 10^6^ (28)	0.11 (12)	25 (15)
RNA-L1a	2.6 × 10^6^ (36)	0.37 (17)	151 (19)	1.8 × 10^6^ (17)	0.22 (6)	123 (11)
RNA-HP2a	8.3 × 10^6^ (9)	0.18 (13)	22 (22)	1.1 × 10^6^ (27)	0.04 (23)	37 (13)
RNA-HP2b	8.0 × 10^6^ (10)	0.27 (33)	33 (28)	2.1 × 10^6^ (11)	0.06 (5.5)	26 (12)

a The coefficient of variation (CV %) is based on three replicates for most interactions except for RRM2 binding to RNA-HP2a and HP2b, which is based on four replicates.

The kinetics of all RRM-RNA interactions were in the same range, with less than a 10-fold variation in the association and dissociation rate constants. There were, however, differences in the affinity. RRM2 apparently had a slightly higher affinity (lower *K*_D_) than RRM1 for interactions with both linear RNAs and one of the double motif structures. For RNA-HP2b, the two RRMs had similar affinities, but the kinetics were different, with RRM1 showing faster association and dissociation rate constants. Replacement of the uracil residue of the UAG binding sequence RNA-L1 by a cytosine (thus generating RNA-L1a) significantly reduced the affinity for both RRMs and resulted in a 3.2-fold weaker affinity for RRM1 and a 4.9-fold weaker affinity for RRM2.

Although both RNA-HP2a and RNA-HP2b have two recognition motifs, the curves were well described by the 1:1 interaction model (Figures 1 and S6–S9). This indicates that the interaction is either dominated by binding to one of the UAG motifs or binding to both UAG motifs with similar kinetics. A close examination of the RRM sensorgrams in Figure 1 reveals that the signal does not return to baseline in some cases, especially after injections at high concentrations. This may be caused by nonspecific binding to the sensor chip and could lead to a slight underestimation of the dissociation rate constants, *k*_d_, and thus also of the *K*_D_-values.

### Musashi-1 Interactions with RNA

**Figure 2:**
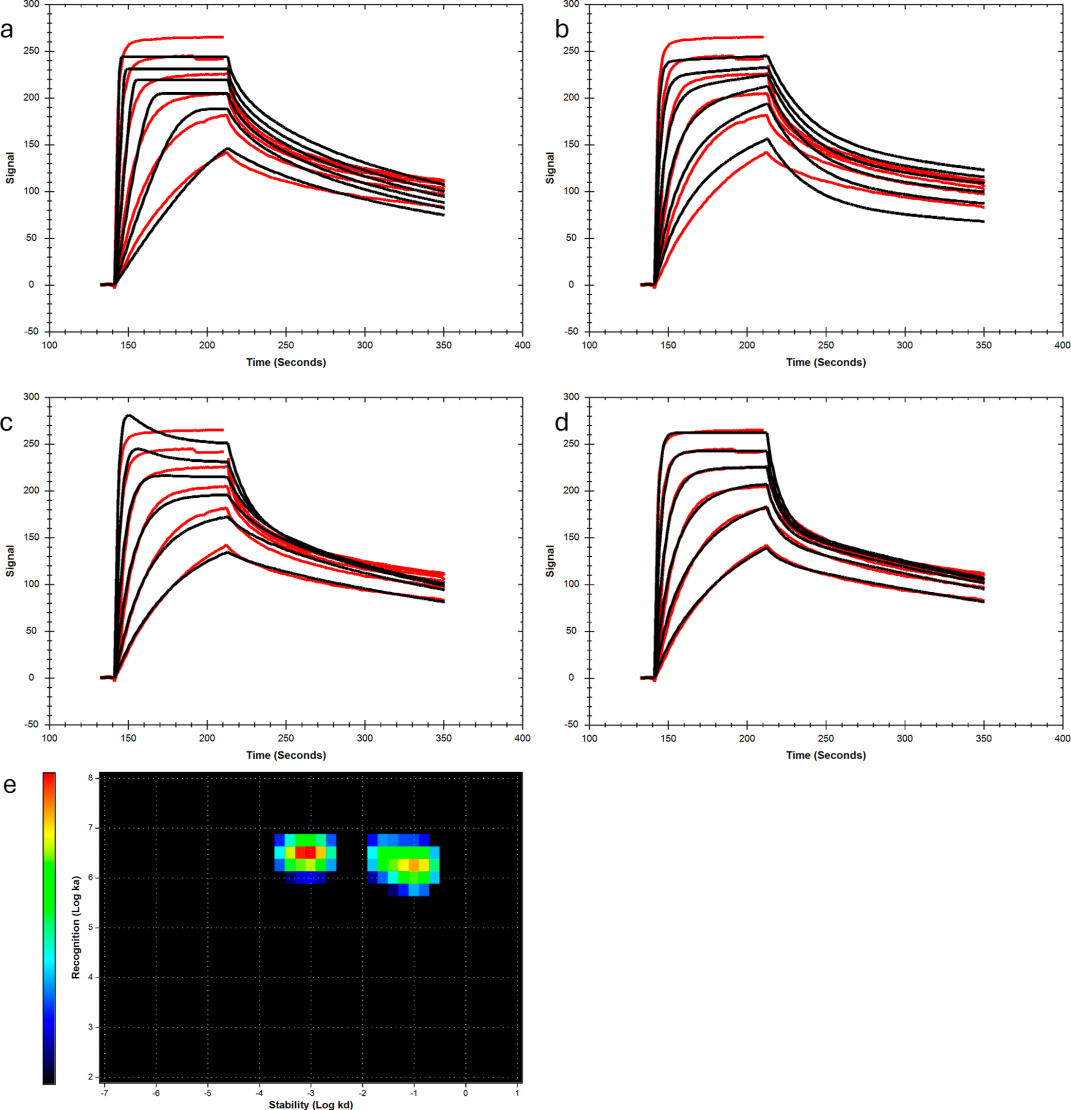
Typical sensorgrams (red) for an interaction between MSI1 (7.8 nM to 250 nM) and RNA-L1 immobilized to 82 RU. Black lines represent curves obtained after regression analysis of experimental data with (a) a 1:1 model (Scheme 1), (b) 1:1 induced fit model (Scheme 4), (c) bivalent analyte model (Scheme 3), and (d) heterogeneous ligand (1:2) model (Scheme 2). (e) InteractionMap analysis of the sensorg*r*ams.

Sensorgrams for the interaction between MSI1 and an RNA containing a single UAG motif (RNA-L1), as presented in Figure 2, display biphasic dissociation. The initial first few seconds of the dissociation are rapid, followed by a much slower dissociation. As RNA-L1 only contains one binding motif and the binding of both isolated RRMs is well described by a 1:1 model and had similar kinetics, a similar 1:1 binding pattern was also expected for MSI1 interaction. However, as can be seen in Figure 2a, the 1:1 model did not fit well, and the resulting χ^2^-values were high (Table 3). Therefore, more complex models were explored to characterize the MSI1 interaction. Sensorgrams of MSI1 interacting with L1-RNA fitted with four different models and InteractionMap analysis are shown in Figure 2a–e. MSI1 interactions with RNA-L1 were also analyzed using Hill slopes to evaluate cooperative effects, but no cooperativity was observed.

**Table 3:** MSI1 Binding to RNA-L1^a^

model	ka1 (M–1 s–1)	*k*_d1_ (s^–1^)	*K*_D1_ (nM)	ka2 (M–1 s–1)	*k*_d2_ (s^–1^)	*K*_D2_ (nM)	χ^2^
1:1	3.7 × 10^7^ (88)	7.2 × 10^–2^ (87)	2 (37)				294
induced fit	2.5 × 10^6^ (87)	6.2 × 10^–2^ (36)	24 (41)	2.5 × 10^–1^ (234)	1.5 × 10^–2^ (182)	8 (39)	277
bivalent analyte (2:1)	1.9 × 10^6^ (84)	4.2 × 10^–2^ (33)	41 (114)	4.4 × 10^4^ (115)	1.9 × 10^–1^ (120)	4441 (23)	204
heterogeneous ligand (1:2)	8.8 × 10^6^ (201)	1.0 × 10^–1^ (40)	58 (59)	2.0 × 10^6^ (72)	3.5 × 10^–3^ (66)	2 (32)	52
rapid 2:1	8.8 × 10^6^ (201)	1.0 × 10^–1^ (40)	58 (59)			1380 (24)	

a Average and coefficient of variation values (CV %) for the association (*k*_a_) and dissociation (*k*_d_) rate constants and affinity (*K*_D_) obtained through applying various models to experimental data. In total, seven replicates with maximum MSI1 signal levels at 250 nM ranging from 70 to 700 RU were evaluated. For the complex models, the first interaction, characterized by *k*_a1_, *k*_d1_, and *K*_D1_, represents the first binding step of MSI1 with RNA. The second process, characterized by *k*_a2_, *k*_d2_, and *K*_D2_, represents the second process, as given by Schemes 2, 3, and 4. For the bivalent (2:1) model, *k*_a2_ and *K*_D2_ values are transformed from signal to concentration units using the 0.22 μM/RU conversion factor. For the induced fit model, *k*_a2_ is given in s^–1^. Presented average χ^2^-values were obtained by dividing χ^2^-values for each replicate by the max signal (mRU) at 250 nM.

### Selection of the Interaction Model

All complex models used for describing MSI1 interactions with the RNA surface account for two binding processes, the heterogeneous ligand model (1:2) for two independent ones (Scheme 2), and the bivalent analyte and induced fit models for two consecutive processes (Schemes 3 and 4). When comparing the goodness of the fit, based on a visual comparison of fitted and experimental curves (Figure 2 and the χ^2^-values in Table 3), it is clear that complex models better fit to the sensorgrams than the simple 1:1 model. As complex models have more parameters that can be varied to give a potentially better fit of the same experimental data, the analysis should include model discrimination procedures to validate the model and exclude other potentially valid models. One of these discrimination procedures is to evaluate the sensitivity of the fitted parameter through the U-values.^30^ U-values indicate how unique the fitted parameters are. U-values and selection criteria are given in the Supporting Information. Visual comparison of the different model fittings was enhanced by presenting the residual plots, which show the difference between experimental and fitted curves (Figures S2–S13 and S15).

The affinities derived from the complex models for the first interaction process (*K*_D1_) resemble those obtained for interactions of RNA-L1 with the individual RRMs (Tables 2 and 3).

The heterogeneous ligand (1:2) model fitted the experimental data best (Figure 2d), indicating the presence of two independent interactions with RNA-L1. The difference in the two interactions is mainly in the stability of the binding process, with the high affinity interaction having a 28-fold slower dissociation rate constant. This result is confirmed by InteractionMap analysis, as shown in Figure 2e, where two distinct 1:1 like interactions were observed, both with a similar on-rate but different off-rates.

The induced fit model (Figure 2b) estimated that the overall affinity became roughly three times higher when taking a stabilization step into account and based on the *k*_a2_/*k*_d2_ ratio that more than 90% of bound MSI1 is in the stabilized conformation at equilibrium. However, the fit was poor, as reflected by a 5-fold higher χ^2^-value than the 1:2 model.

When applying the bivalent analyte model, the curves fit the dissociation better than the 1:1 and induced fit models, but the fits were overall poor, also reflected by a relatively high χ^2^-value and poor U-values (Tables S10–S13). However, the variation in *K*_D2_ was low between replicates, and the average value suggests that a bivalent complex is formed with a low μM affinity.

### Discrimination Between Complex Models Using Extended Experiment Design

**Figure 3:**
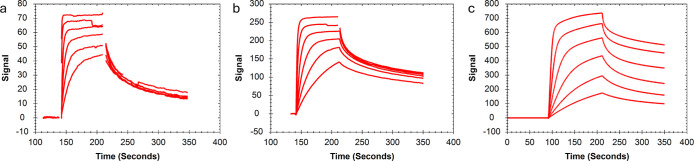
Sensorgrams depicting MSI1 interacting with RNA-L1 immobilized to different levels, representing different sensor surface densities, (a) 39 RUs, (b) 82 RUs, and (c) 245 RUs.

Although a good fit was obtained with the heterogeneous ligand (1:2) model (Figures 2d and S10–S13), it cannot be assumed that it accurately describes the interaction mechanism without further experimental proof or a reasonable mechanistic model. An inconsistency with this model was found since the contribution of the rapid interaction was higher for low-density RNA surfaces, and the dissociation of MSI1 was slower from the high-density RNA surface (Figure 3). This behavior is expected for bivalent interactions formed with two proximal RNA strands on the sensor chip. As RRM interactions with RNA are rapid (Table 2), it is likely that MSI1 also rapidly interacts with RNA, as supported by the fast rate constants of the first interaction obtained from all complex models. MSI1 is initially bound monovalently, after which it can either dissociate rapidly or rapidly engage in a bivalent interaction. A pseudoequilibrium situation is created, for which the transition from mono- to bivalent is hardly visible in the experimental data. For this pseudoequilibrium situation, the two interactions obtained by applying the heterogeneous ligand model describe the monovalent interaction and a second interaction that has a similar association rate constant and a slow dissociation. The second interaction reflects the complete dissociation of the bivalently bound MSI1. This means that the affinity (*K*_D2_) and rate constants (*k*_a2_ and *k*_d2_) of the second interaction describe the bivalent binding of MSI1 from solution to two RNA molecules instead of the interaction with a different binding site (2nd step in Scheme 2) or the second step of the bivalent process (Scheme 3).

To simplify interaction patterns and avoid bivalent interactions that involve two molecules on the sensor chip, low immobilization levels are generally used. However, for the MSI1-RNA-L1 interaction, bivalent complexes were readily formed and were clearly present at the lowest immobilization levels used. This is seen from the dissociation curves that align after a few seconds, independent of the concentration used. Thus, monovalently bound MSI1 either rapidly dissociates or engages in a pseudoequilibrium of bivalently bound complexes (Figure 3a). Using even lower immobilization levels would compromise the signal quality. Therefore, to extract the *K*_D_-values of the second interaction (Scheme 3) from values generated by applying the 1:2 model, a new approach was taken.

### Estimation of *K*_D_-Values for Fast Bivalent Interactions

**Figure 4:**
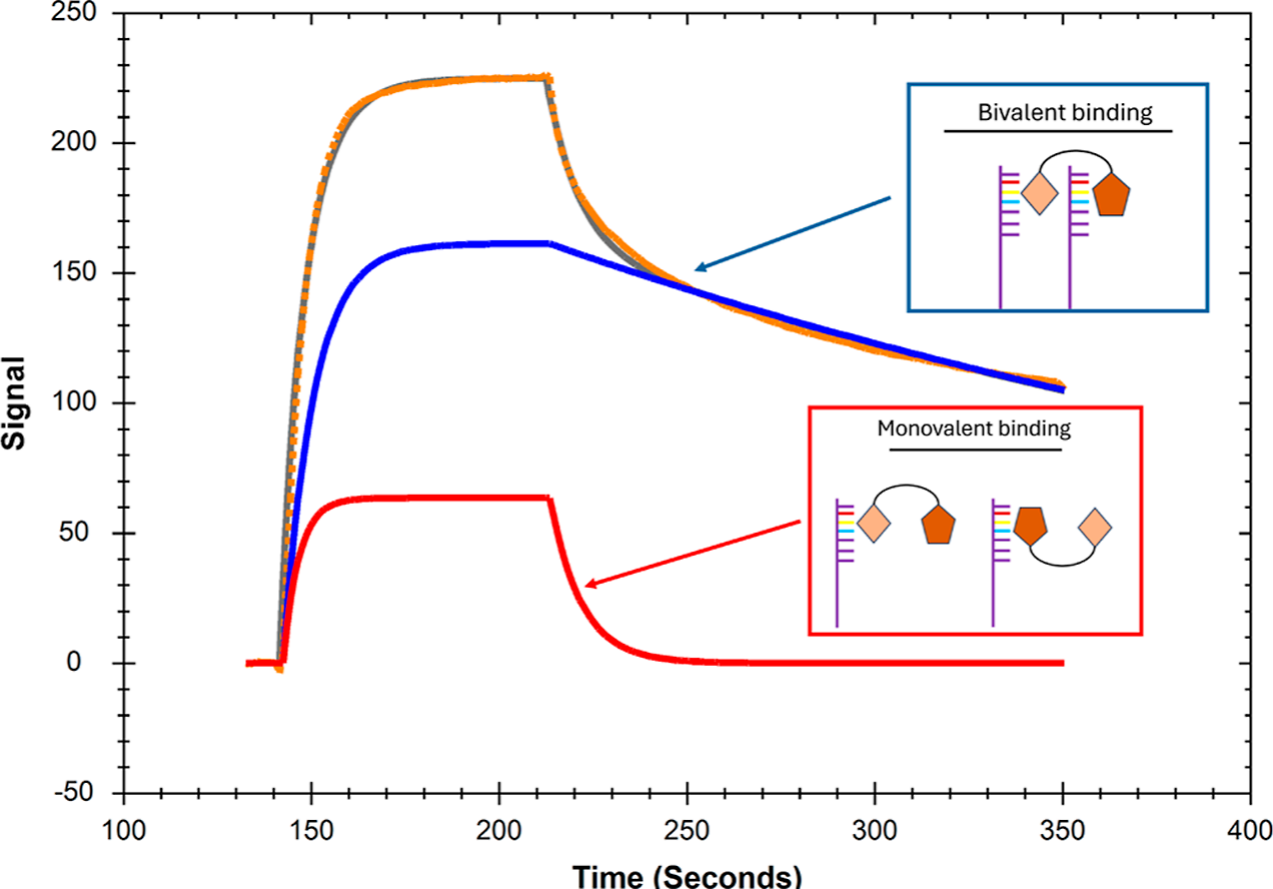
Sensorgram of the MSI1 interaction with RNA-L1 at 62.5 nM (dark gray), and results from applying heterogeneous ligand (1:2) model fit (in dotted orange line) depicting the rapid dissociating, monovalent, interaction (red), and slow dissociating interaction corresponding to bivalently bound MSI1 (blue).

To overcome the experimental and data fitting challenges of the bivalent interactions observed for MSI1 and RNA, we developed a new method to estimate the two equilibrium constants (*K*_D1_ and *K*_D2_) for a fast bivalent interaction mechanism (Scheme 3 and eq 3) from values obtained from the heterogeneous ligand (1:2) model. In Figure 4, the sensorgrams for the interaction of MSI1 at 62.5 nM with RNA-L1 are shown (top trace, dark gray in Figure 4). Fitting the 1:2 model to the sensorgrams reveals that the data is a combination of a rapidly dissociating fraction (bottom trace, red) and a slow dissociation fraction (middle trace, blue). *K*_D1_ can be estimated from traces representing the monovalent interaction.

For the second interaction, the later phase of the dissociation is slower as it represents complete dissociation of bivalently bound MSI1 and thus depends on both dissociation rates, *k*_d2_ and *k*_d1_, and rebinding from a monovalent state into a bivalent state with rate constant *k*_a2_. As only monovalently bound MSI1 can dissociate, the observed dissociation rate constant (*k*_obs_) in this later phase of dissociation depends on the fraction of monovalently bound analyte relative to the total amount of bound analyte. This fraction of monovalently bound MSI1 dissociates with the rate constant *k*_d1_ and is represented by *k*_d2_ from the 1:2 model (eq 4).

When eq 4 is rewritten into eq 5, a relationship between both dissociation rates obtained from the 1:2 model (*k*_d1_ and *k*_d2_) and the ratio between monovalent and bivalent species at equilibrium is obtained.
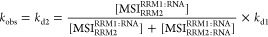
4
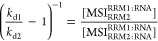
5

The affinity of the bivalent interaction is described by the ratio between the concentrations of unbound and bound species (eq 6). As the *k*_d1_/*k*_d2_ ratio can be used to calculate the ratio between the concentrations of mono- and bivalently bound MSI1 using eq 5, only the concentration of unbound RNA on the sensor chip is required to calculate the affinity, *K*_D2_. The free RNA concentration can also be extracted from the 1:2 fit, as the model estimates the maximum signals (*B*_max_) when all RNA is bound by MSI1 for the mono- and bivalently bound fractions. Using the 0.22 μM/RU conversion factor, the total RNA concentration on the sensor chip can thus be estimated. The signal at the later phase of dissociation represents the sum of the monovalent (*B*_obs1_) and bivalent (*B*_obs2_) fractions for which the ratio is given by eq 5. The difference between the *B*_max_ values from the 1:2 model and the average of the signals for the same interactions during the last 30 s of dissociation was used to estimate the free RNA concentration. Considering that bivalently bound MSI1 binds two RNA molecules, the free RNA concentration at the later phase of dissociation was calculated, as given by eq 7.
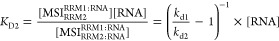
6With
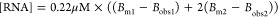
7

For MSI1 interactions with RNA-L1 shown in Figure 2, the dissociation rate stabilizes 28-fold, meaning that the *k*_d2_/*k*_d1_ ratio, and thus the ratio between monovalent and bivalently bound MSI1, amounts to 3.46 × 10^–2^. The unbound RNA concentration, estimated from average *B*_max_ values and signals at the last 30 s of dissociation, amounted to 226 RU and thus corresponds to 5.77 × 10^–5^ M according to eq 6. The resulting affinity for the bivalent binding thus amounted to 1.70 μM and is 3-fold weaker than the one obtained from the bivalent model, while the affinity for the first, monovalent binding step is similar to that derived from the bivalent model and those observed for RRM1 and RRM2 (Tables 2 and 3). The rate constants for the monovalent interaction derived with the new method differ roughly 2-fold from the values obtained for RRM1 and RRM2, but this difference is less than the rate constant obtained from the bivalent model.

**Figure 5:**
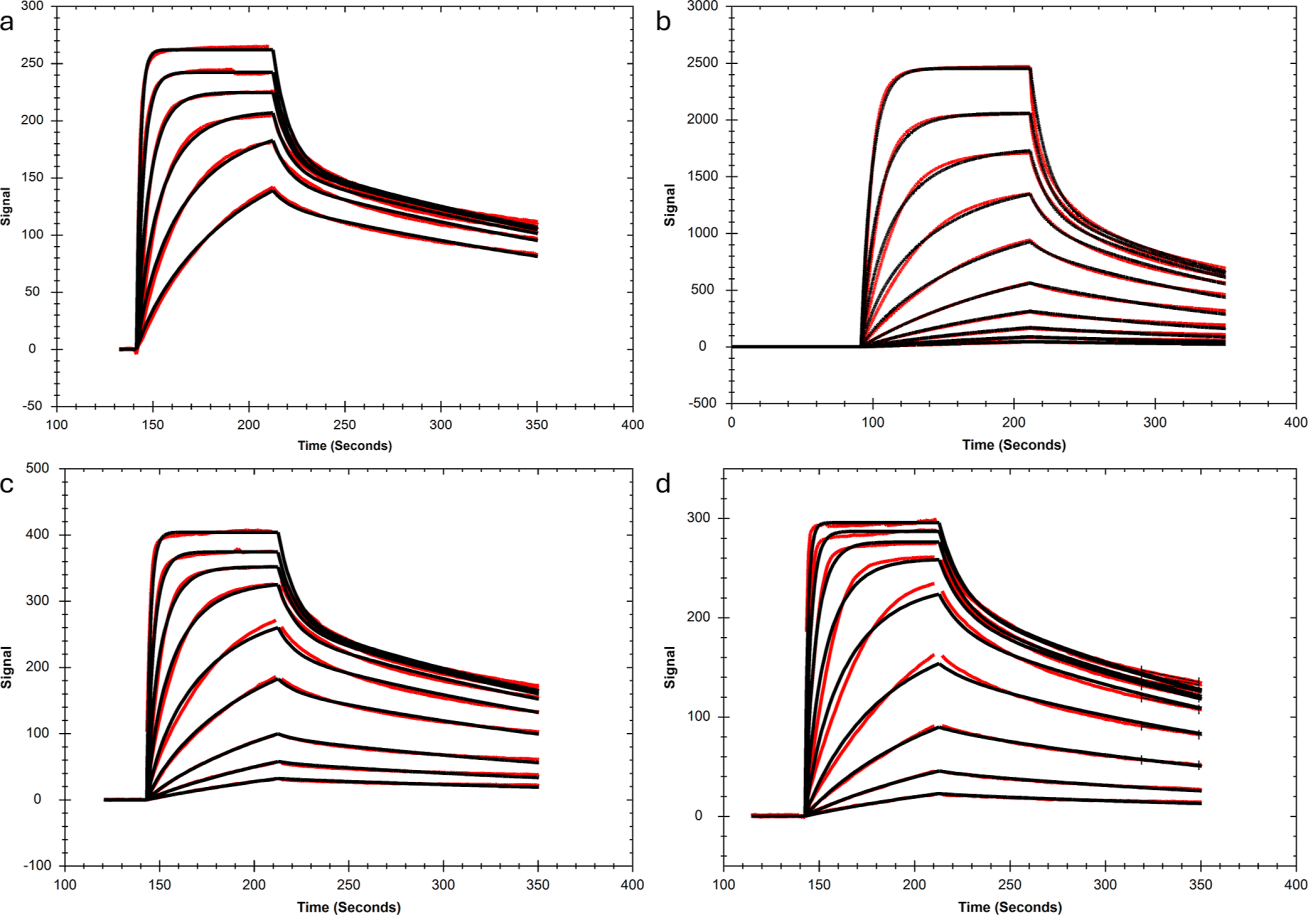
Representative sensorgrams (red) for MSI1 interactions with four different RNA strands: (a) RNA-L1 (b) RNA-L1a, (c) RNA-HP2a, and (d) RNA-HP2b. For extracting information on the bivalent interaction process using the new method, all sensorgrams were fitted with a heterogeneous ligand (1:2) model (black). The concentration ranges for the following interactions were: MSI1 to RNA-L1 7.8 to 500 nM; MSI1 to RNA-HP2a and HP2b 1.9 to 500 nM; and MSI1-RNA-L1a 1.9 nM to 1 μM.

All MSI1 interactions with other RNAs (Figure 5) displayed similar interaction patterns and similar dependence on the RNA surface concentration. Therefore, the new method was applied to all MSI1 - RNA sensorgrams. Affinities for the mono- and bivalent interactions, as obtained from both the bivalent model and the new method, are presented in Table 4, together with the rate constant as derived from the new method. Compared to the bivalent model, the new method resulted in better fits with roughly 4-fold lower χ^2^ values and less variation between replicates, as seen from the CV values presented in Table 4. The affinity values obtained from both methods, however, were in the same range.

**Table 4:** Kinetic and Affinity Data for MSI1 Interactions with Different RNA Strands^a^

RNA strand	new model *K*_D2_ calculation				bivalent *K*_D2_	
	*k*_a1_ (M^–1^ s^–1^)	*k*_d1_ (s^–1^)	*K*_D1_ (nM)	*K*_D2_ (μM)	*K*_D1_ (nM)	*K*_D2_ (μM)
RNA-L1	8.8 × 10^6^ (201)	0.10 (40)	58 (59)	1 (24)	42 (114)	4 (23)
RNA-L1a	7.6 × 10^4^ (14)	0.09 (9)	1170 (19)	60 (4)	1410 (91)	87 (57)
RNA-HP2a	1.6 × 10^6^ (11)	0.12 (7)	77 (13)	3 (10)	45 (104)	2 (141)
RNA-HP2b	1.9 × 10^6^ (9)	0.09 (11)	49 (20)	3 (8)	144 (67)	4 (69)

a Rate constants of the monovalent interaction (*k*_a1_, *k*_d1_) were obtained from applying the new method, while affinity values for both the mono and bivalent interactions (*K*_D1_ and *K*_D2_) were estimated using both the new method and the bivalent model. Presented values are averages of triplicates; CV values (%) are shown in parentheses.

### Musashi-1 Interaction with RNA-L1a

The association rate constant for the interaction between MSI1 with RNA-L1a is more than 100-fold lower than for the interaction with RNA-L1. Interestingly, the association rate constant for MSI1 with RNA-L1a was also more than 100-fold slower compared to the binding of the individual RRMs that have *k*_a_ values of 2 × 10^6^ M^–1^ s^–1^. This is in contrast to the pattern observed for RNA-L1, where the association rate constants of the RRMs and MSI1 were similar. The dissociation rate constants obtained from applying the 1:2 model showed that dissociation of bivalently bound MSI1 was 20-fold slower than that of monovalently bound MSI1. As a result, the calculated *K*_D2_ was 60 μM and thus 40 times weaker than the bivalent interaction with the RNA-L1 strand. A similar affinity value for the bivalent interaction was obtained from the bivalent model (Tables 4 and S10).

### Musashi-1 Interaction with RNA-HP2a and RNA-HP2b

Both RNA-HP2a and RNA-HP2b have two UAG motifs, which, in principle, allow for intramolecular bivalent binding. However, the sensorgrams had similar curvatures as those for the linear single motif RNA strands with a rapid slowing of the dissociation rate, which is dependent on the levels of immobilized RNA. This indicates that the slowing of the dissociation rate is dominated by intermolecular bivalency rather than intramolecular bivalency. InteractionMap analysis confirms the presence of two different binding events with similar *k*_a_-values with a clear peak for a rapidly dissociating fraction and another peak for the slower dissociating fraction (Figure S14).

Analysis of binding of RNA-HP2a and RNA-HP2b to MSI1 revealed two interactions with similar association and dissociation rates for the monovalent interaction. Compared with the RRMs, association rates are slower, leading to a weaker affinity for the monovalent MSI1 interactions. The affinities for the second, bivalent interaction are highly similar, and the interaction is roughly 2-fold weaker compared to the linear single motif RNA-L1.

## Discussion

Injecting a truncated variant of human MSI1 encompassing RRM1 and RRM2 in tandem to various immobilized RNA strands resulted in sensorgrams showing that there is an initial rapid dissociation that is comparable to the rapid dissociation of individual RRMs, but that it is later considerably slower. There are consequently two fractions of MSI1 that bind with different stability. Since this was observed for the single UAG motif containing RNA-L1, especially for high-density surfaces, it can be inferred that stabilization of MSI1 binding is caused by bivalent binding to different RNA strands on the sensor chip rather than dual motifs in a single strand. Our data do not exclude that MSI1 may also bind bivalently to a single RNA strand encompassing two binding motifs, but such an intramolecular mechanism would be described by an induced fit model, which fitted the experimental data poorly (Figure S15 and Table S13).

Bivalent interactions are typical when an immobilized ligand interacts with two analytes on a sensor surface, as, for example, reported for HRG1β binding to HER3,^35^ SARS-CoV-2 spike protein binding to the ACE2 receptor,^36^ antibody–antigen binding processes,^37^ and for other RBP-RNA interactions.^32^

Although we could easily conclude that the interaction was bivalent, when using the bivalent model for nonlinear regression analysis of the sensorgrams, the fits and the obtained parameters, as observed from the U-values, were poor, and χ^2^-values were relatively high. An exploration of the starting estimates gave different results, indicating the presence of multiple local minima. By using interaction rates close to those observed for the individual RRMs as starting estimates, more reproducible and accurate results were obtained, but χ^2^-values remained roughly 4-fold higher than the ones obtained when applying the heterogeneous model. From a visual comparison between experimental and fitted curves, it was seen that it was the rapid transition from a quick initial dissociation to a much slower dissociation in just a few seconds that made it difficult to use the bivalent model to fit the data. The difficulty in applying a bivalent model to complex data has previously been addressed,^28^ and fast bivalent interactions have instead been analyzed using less common data evaluation methods, such as cooperativity analysis^31−33^ and advanced data analysis algorithms like InteractionMap.^35,36^ When the bivalent model has been used, the quantification of rate constants and affinities was not mentioned.^38^

Seeing that stabilization of the binding via intermolecular interactions on the sensor chip is dependent on the RNA surface coating density, kinetic and affinity values derived from models that only take interactions with single immobilized molecules into account are useful only when quantifying RNA and MSI1 surface concentrations. To overcome the challenges with applying the bivalent model for analysis of the data, the new method proposes estimation of *K*_D2_ values in a manner that is independent of the RNA surface concentration and is based on parameters obtained from fitting using the heterogeneous ligand (1:2) model. This model has previously been used to quantify avidity enhancement by using the ratio between the fast and slow dissociating fractions^37^ and fitted the data better than the bivalent model. Although it is theoretically suboptimal, it provides estimates of the rate constant and affinity of the monovalent interaction (*k*_a1_, *k*_d1_, *K*_D1_), the stabilization due to bivalent interactions (*k*_d2_/*k*_d1_), the relation between monovalent and bivalent complexes together with the RNA concentration, and thereby the affinity of the bivalent interaction (*K*_D2_). Importantly, the resulting *K*_D2_ values were comparable to those obtained from the bivalent model, but the accuracy was higher, as indicated by almost 4-fold lower χ^2^ values and lower variation between replicates. As the number of parameters to be fitted is similar for both models, experimental data are better described by two individual interactions (1:2 model) than a coupled bivalent interaction model (2:1 model).

By design, this new method of analyzing the bivalent interaction on the sensor chip is not able to extract rate constants for the bivalent interaction step. However, from the curve shape, it is obvious that rapid (re)binding of monovalently bound MSI1 to another RNA strand on the chip strongly reduces dissociation of MSI1 from the chip once the unbound RNA concentration becomes sufficiently high. Intermolecular bivalent interactions take place between two molecules that are bound to the sensor chip and thus are both restricted in their lateral and rotational mobility. It is therefore likely that the actual association rate, and thus the affinity, of this bivalent interaction in solution is higher. Nevertheless, the accuracy of this method provides a means to evaluate the impact of the sequence and structure of various RNA strands on their ability to be captured in a bivalent manner by MSI1. An alternative approach based on multiple starting estimates for the parameters that are varied in the fitting process so that local minima and inaccurate estimation of some of the interaction parameters were proposed by Nguyen et al.^28^ Combining both approaches has good potential to improve the analysis of intermolecular interactions.

### Limitations of the Method

The calculation of the affinity corresponding to the second interaction in a bivalent binding (*K*_D2_) requires the data to be suitable to be fitted with a 1:2 model. Consequently, the new method loses accuracy when the experimental data is strongly dominated (>90% of the observed interaction) by either the monovalent interaction at extreme low RNA immobilization densities or when MSI1 predominantly forms bivalent complexes at highly coated surfaces. Another limitation is that the new method uses the parameters from the 1:2 model without correcting for mass transport limitations as used for analyzing interaction patterns of RRMs according to Scheme 1. However, analyzing the MSI1 interaction data while including the mass transport rate constant (*k*_t_) in the fitting process strongly increased the variation between replicates for the rate constants. The affinity values for the monovalent and bivalent interaction were only slightly affected.

### RRMs and MSI1 Interactions with Single Motif RNAs

It has previously been proposed that RRM1 and RRM2 play different roles in RNA binding, with RRM1 guiding the recognition and RRM2 increasing the stability of the complex, but experimental data from RRM2 binding was lacking.^22^ In the present study, such a difference was not observed as both RRMs interact with highly similar association rate constants and thus similar recognition for the UAG motif in RNA-L1. Only the dissociation rate was somewhat (less than 2-fold) slower for RRM2, resulting in a higher *K*_D_ for RRM2 compared to RRM1. The previously published *K*_D_-value for the RRM1 interaction with a single motif RNA, obtained through fluorescence polarization, was 68 ± 3 nM,^22^ which aligns well with the data we obtained using a SPR-based biosensor. For MSI1-RNA-L1, the new method predicts an affinity for the monovalent interaction that is somewhat weaker than that observed for the individual RRMs. Previous kinetic studies of MSI1 interactions with RNA using another biosensor-based technology^25^ resulted in *k*_a_ values of 0.7 to 2.6 ·10^7^ M^–1^ s^–1^, and *k*_d_ values of 0.01 to 0.1 s^–1^, with *K*_D_-values ranging from 0.6 to 8 nM. Dolcemascolo et al. used a 1:1 model for the analysis, although some of the sensorgrams showed a biphasic behavior that suggested that the interaction was more complex. Nevertheless, values for the monovalent interaction are in the same range as the ones presented in this study and values that have been published earlier,^22^ with apparent affinities in the low nanomolar range. Affinities for the bivalent interaction have not previously been quantified. The new method calculated a *K*_D_ of 1.4 μM for the bivalent interaction and thus more than 20-fold weaker than the monovalent interaction. This value may be an underestimation of the affinity in solution since bivalent interactions occur between two RNA strands that are both tethered to the polymeric matrix on the sensor chip. It restricts lateral and rotational movements that are possible in solution, where the RNA is not tethered to a surface.^39^

Ruth Zearfoss et al. also demonstrated that substitutions in the UAG motifs have a profound effect on the affinity for RRM1 with a 6-fold reduction and MSI1 with a 16-fold reduction. This is supported by the present study, where substitution of the first uracil of the UAG sequence by a cytosine clearly affected the binding kinetics and affinity. The affinity was reduced 3.2-fold for RRM1 and 5-fold for RRM2, and this weaker affinity is primarily caused by a lower stability of the RRM1-RNA complex. For the interaction between MSI1 and the CAG motif containing RNA-L1a, the substitution has a much stronger effect with a 20-fold weaker affinity of the monovalent binding and a 43-fold weaker bivalent binding affinity. This variation in affinities is in line with previously reported binding modes for RBPs, for which the binding is usually driven by several multivalent interactions instead of a high affinity for a single specific sequence.^40^ Nevertheless, no binding was observed for the negative control that contains CG and CCG motifs; therefore, the presence of adenine in the UAG motif appears to be crucial for the binding to occur.

RRM binding to RNA-HP2a and HP2b, both harboring two binding motifs, showed a similar behavior, well described by a 1:1 model. This means that there is either no significant difference in the interactions with the two binding motifs or that one of the binding motifs is inaccessible, for example, due to its location in the hairpin structure. Also, MSI1 interactions with both RNA-HP2a and HP2b are similar with respect to interaction patterns and resulting *K*_D_-values. Binding of MSI to RNA-HP2a and HP2b could theoretically be intra- or intermolecular as each RNA has two UAG binding motifs. Intramolecular binding would be characterized by a stabilization of the interaction that is independent of the RNA immobilization density due to rapid rebinding to the second UAG motif, but this is not observed in the present data. The preference to form intermolecular interactions over intramolecular binding may be explained entropically since intramolecular interactions would limit the possible conformations by forcing a less flexible binding between two near sequences. In contrast, intermolecular binding would offer more entropically favorable binding conformations. For MSI1, it has been reported that it is flexible enough to allow interactions in between RNA sequences separated by a variable number of nucleotides (between 1 and 50).^41^

The intermolecular bivalent binding mechanism also explains why the stable interaction dominates at low concentrations of MSI1, where relatively few oligomers become occupied by MSI1 and thus capable of forming a bivalent complex. The opposite applies when injecting high concentrations of MSI1. Upon dissociation, more and more RNA strands become available for bivalent binding, and eventually a state is reached where binding is equally stable regardless of the injected concentration, as depicted in Figure 2. This complex stability would thus represent the capability of MSI1 to stabilize its interaction with RNA-coated sensor chips by rapid rebinding upon dissociation of one of the two RRM domains prior to complete dissociation.

As RNA-HP2a has guanine in front of both UAG motifs while this is an uracil residue in RNA-HP2b, the similar affinity of MSI1 for these motifs confirms the observation that RRMs primarily interact with the three-nucleotide UAG motif^22^ and not with the longer (G/A)U_1–3_AGU motif that has been proposed earlier.^21^ It has been described that RRMs can bind with high affinity to hairpin structures.^42^ Both RNA-HP2a and HP2b have the potential to form hairpins, and RRMs show a relatively high association rate constant, indicating a possible role of the hairpin in improving the recognition of the binding motif by RRMs.

When affinities between linear and hairpin UAG motif-containing RNAs are compared, it is observed that the affinity for the monovalent interaction is similar and that the affinity of the bivalent interaction is 2-fold weaker for the double motif RNAs. Thus, both the presence of a second UAG motif and a more rigid secondary structure of the RNA motifs do not seem to contribute to a stronger affinity as has been reported previously for RBP RNA interactions.^40^

Multiple studies have pinpointed the importance of cooperativity, multivalency, and avidity in RNA-protein interactions,^31,33^ with all these concepts being usually indistinctly used. Cooperativity is described as an interaction process where the binding of the first domain is followed by conformational changes that modify the other domains and therefore increase the affinity for subsequent binding events. To measure the degree of cooperativity in a certain binding event, the Hill coefficient has been widely used, which refers to the minimal number of domains that interact in the system.^32,43^ Cooperativity, however, was discarded as a mechanistic option for the MSI1 interactions studied here, as the obtained Hill coefficients indicated negative cooperativity rather than positive cooperativity.

A potential biological effect of multivalent systems is the establishment of higher local concentrations, or effective concentrations.^44^ A higher effective concentration can occur when one of the RRMs rebinds before the second one dissociates and when this rebinding is relatively fast compared to the diffusion of interacting components. From the interaction curves, it is obvious that MSI1 is efficient in forming intermolecular interactions and is effectively retained in the 200 nm thick polymer layer on the sensor chip where RNA is immobilized. A high local concentration translates into an increased fraction of bound species for interactions with a certain affinity, as can be inferred from eq 3, e.g., a 2-fold higher concentration of both interaction partners will lead to a 4-fold higher concentration of the bound complex.

## Biological Implications of Fast, Multivalent Binding

To understand the binding process in relation to its biological activity, focus has traditionally been on affinities, as they describe the fraction of bound molecules as a function of the concentration of interacting molecules when the binding process is in equilibrium. However, in living systems, concentrations of interacting molecules are constantly changing, and information on the dynamics of the binding process therefore becomes important for understanding biological function.^45^ For drug optimization, focus has been on dissociation rate constants.^24,46,47^ But more recently, rapidly associating ligands have been found to represent an interesting aspect of interaction dynamics with respect to drug efficacy, as it explains the importance of rebinding to the same or surrounding targets before drifting away.^48^ In this study, the binding between both RRM domains and RNA sequences containing the UAG motif was rapid, while the binding curves of the MSI1 construct showed that the binding is strongly stabilized compared to the binding of the individual RRMs. Apparently, MSI1 efficiently binds two sequences on the sensor chip, even when RNA immobilization levels are relatively low. This was further emphasized when the UAG was replaced by a CAG motif in the linear RNA, resulting in roughly 4-fold weaker affinity for the RRMs. However, for MSI1, the affinities for the monovalent and bivalent interaction steps were 20- and 40-fold reduced, respectively. It might well be that this binding behavior reflects the binding processes in cells, where the two RRM domains rapidly bind two RNA strands and efficiently rebind to the same or other RNA strands upon dissociation before drifting away. In this way, a microenvironment could be created with an increased MSI1-RNA concentration that will enhance the biological effect, as higher concentrations will result in a higher bound versus unbound ratio.

This microenvironment formation matches with the previously described MSI1 contribution to the formation of membraneless compartments as stress granules.^20^ Although not included in the truncated version used in the present study, full-length MSI1 contains a disorder tail, and IDRs have been related to phase-separation phenomena in RBPs,^49^ including MSI1.^50^

RNA concentration mediates the formation of cellular condensates by hnRNPA1A, a RRM that is structurally highly similar to MSI1 with two RRM domains and an IDR. At lower RNA concentrations, the condensates showed a dynamic behavior that gradually becomes more static when RNA concentration increases.^51^ A similar process was observed in the data we obtained at different RNA concentrations, with faster observed dissociation at lower RNA coatings, while at higher RNA concentrations, the observed dissociation was slower. Further experiments would be needed to confirm the suitability of our experimental design and analysis to predict the formation of cell condensates.

In conclusion, the importance of multivalent binding in forming RBP-RNA cellular condensates has been widely shown,^45,51,52^ and MSI has been previously described to participate in this process.^19,20,53^ The obtained kinetic profile, the previously explained fast rebinding effect, and the new method to calculate the bivalent affinity perfectly match with the protein participation in these cellular condensates.

## Supporting Information Available

Additional sensorgrams for all individual replicates, residuals plots and tables with kinetic and affinity parameters for each replicate, including U-values, and negative control sensorgrams (PDF)

## Notes

MSI1: O43347 (Uniprot)
