# Supplementary material for: Unraveling the Bivalent and Rapid Interactions Between a Multivalent RNA Recognition Motif and RNA: A Kinetic Approach
Source: Biochemistry. 2024 Oct 14;63(21):2816–29. doi: 10.1021/acs.biochem.4c00301 (PMC11542179; doi:10.1021/acs.biochem.4c00301)
Supplement: Supplementary file 1 — bi4c00301_si_001.pdf [file bi4c00301_si_001.pdf]

Supplementary Information

Unraveling the bivalent and rapid interactions between a multivalent RRM and RNA: a kinetic approach

Guillermo Pérez-Ropero, Anna Pérez-Ràfols, Tommasso Martelli, U. Helena Danielson, Jos Buijs

Sensorgram of MSI1, RRM1 and RRM2 binding to negative control RNA-C

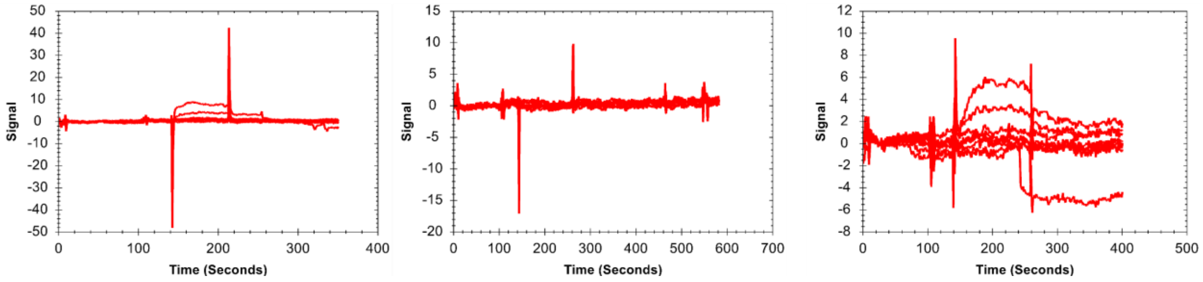

Figure S1: Binding sensorgrams to negative control RNA-C. From left to right: MSI1 (7.8 to 250nM); RRM1 (1.9nM to 500nM) and RRM2 (1.9nM to 500nM).

Sensitivity of fitted parameters: U-value

The U-value estimates the uniqueness of the fitted parameters or parameter-pairs by finding possible correlation between them. The U-value, expressed as a percentage, reflects how much a parameter or parameter-pair can change without significantly changing the goodness of the fit. This is described in detail in Onell & Andersson, 2005.

Therefore, a high U-value means a low accuracy for the parameter or parameter pair obtained from the fitting process, while a low U-value indicates that the parameter is uniquely defined. Parameters with low U-values are not sensitive to variations in data selection and fitting conditions such as starting guesses.

For Biacore systems, a U-value lower than 15 is considered optimal (e.g less than 15% of variation), while a U-value above 25 indicates a correlation between parameters that doesn't allow to uniquely determine the parameter value. The U-value for the parameter or parameter-pair with the lowest accuracy is presented in all supplementary Tables.

Sensorgrams of RRM1 and RRM2 binding to RNA L1, L1a, HP2a and HP2b with residuals plot

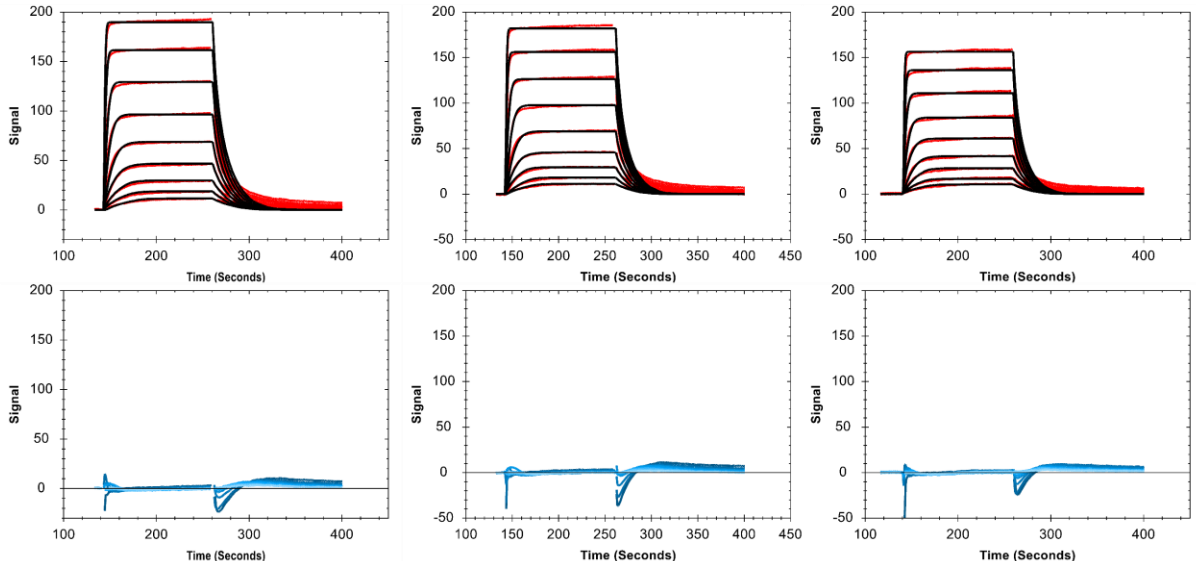

Figure S2: RRM1 binding to RNA-L1. From left to right, replicates 1 to 3. From top to bottom, sensorgram fitted with a 1:1 model, and residuals with concentrations depicted in a blue-gradient where a darker-color implies a higher concentration.

Table S1: Kinetic and affinity values for the interaction between RRM1 and RNA-L1 corresponding to a 1:1 model fitting. Note that Chi^2 values are not corrected by the maximum signals as in the manuscript.

Replicate number	k_a (M^{-1} s^{-1})	k_d (s^{-1})	K_D (nM)	k_t (Signal $M^{-1}s^{-1}$)	Chi^2 (Signal^2)	U-value:
1	3.12 10^6	1.42 10^{-1}	45.4	3.71 10^8	17.58	<0.1 k_d/k_t (%)
2	3.73 10^6	1.81 10^{-1}	48.4	4.00 10^8	20.19	4.7 k_a/k_d (%)
3	3.74 10^6	1.77 10^{-1}	47.4	3.67 10^8	17.36	<0.1 k_a/k_d (%)
Average	3.5 10^6	0.167	47.1			
CV (%)	8.2	11	2.6			

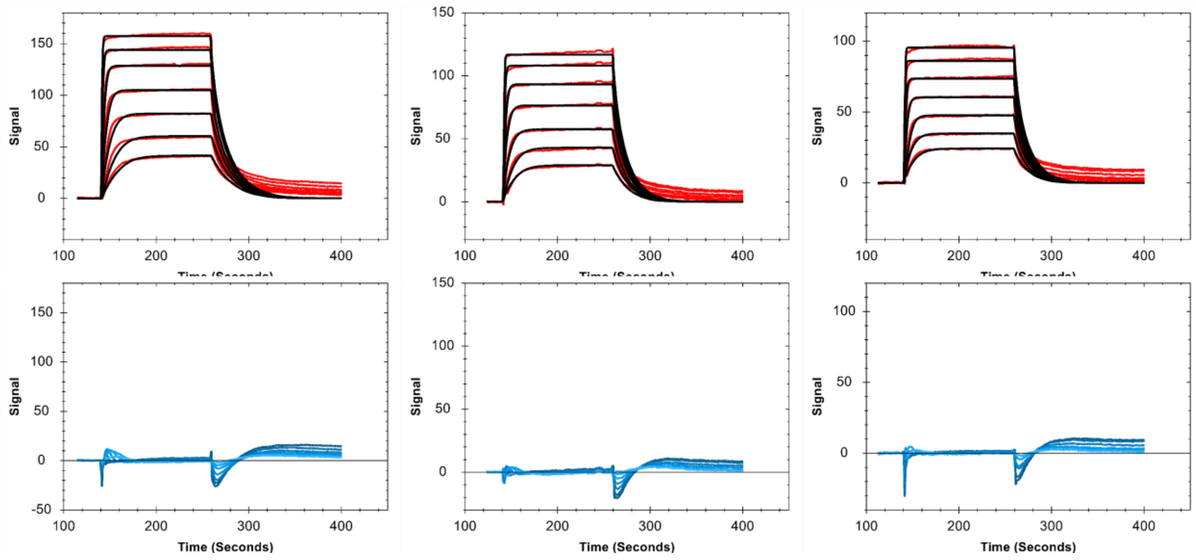

Figure S3: RRM2 binding to RNA-L1. From left to right, replicates 1 to 3. From top to bottom, sensorgram fitted with a 1:1 model, and residuals with concentrations depicted in a blue-gradient where a darker-color implies a higher concentration.

Table S2: Kinetic and affinity values for the interaction between RRM2 and RNA-L1 corresponding to a 1:1 model fitting. Note that Chi^2 values are not corrected by the maximum signals as in the manuscript.

Replicate number	k_a (M^{-1} s^{-1})	k_d (s^{-1})	K_D (nM)	k_t (Signal $M^{-1}s^{-1}$)	Chi^2 ($Signal^2$)	U-value:
1	3.67 10^6	9.85 10^{-2}	26.8	4.73 10^8	43.27	22.6 k_a/k_d (%)
2	3.78 10^6	1.07 10^{-1}	28.3	5.19 10^8	20.43	16.3 k_a/k_d (%)
3	6.48 10^6	1.29 10^{-1}	19.9	4.73 10^8	18.23	26 k_t (%)
Average	4.6 10^6	0.112	25.0			
CV (%)	28	12	15			

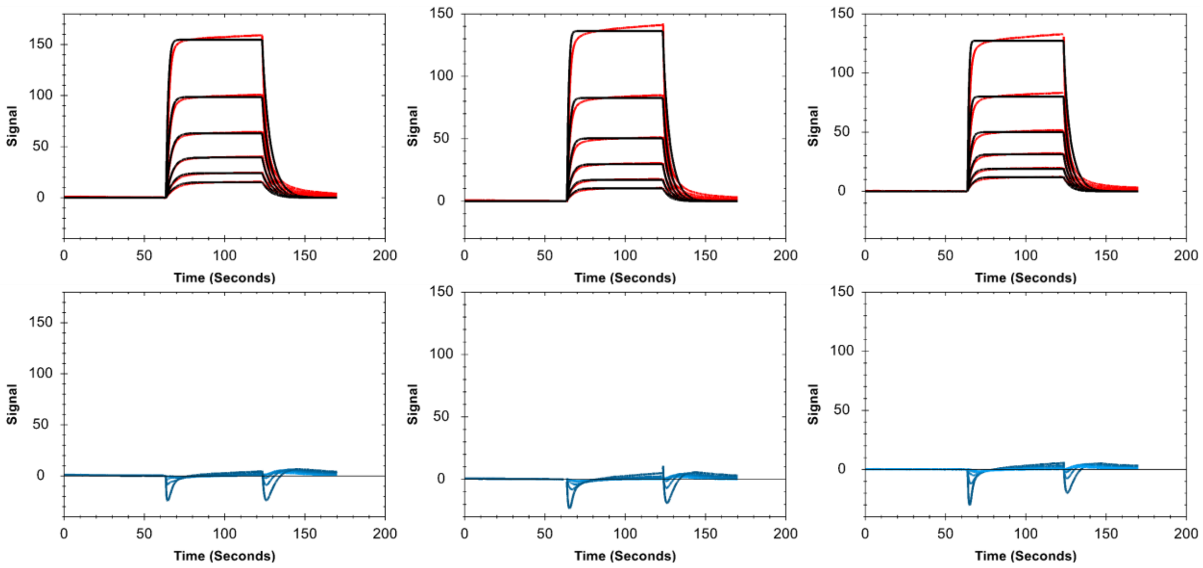

Figure S4: RRM1 binding to RNA-L1a. From left to right, replicates 1 to 3. From top to bottom, sensorgram fitted with a 1:1 model, and residuals with concentrations depicted in a blue-gradient where a darker-color implies a higher concentration

Table S3: Kinetic and affinity values for the interaction between RRM1 and RNA-L1a corresponding to a 1:1 model fitting. Note that Chi^2 values are not corrected by the maximum signals as in the manuscript.

Replicate number	k_a (M^{-1} s^{-1})	k_d (s^{-1})	K_D (nM)	k_t (Signal $M^{-1}s^{-1}$)	Chi^2 ($Signal^2$)	U-value:
1	1.55 10^6	2.84 10^{-1}	183	4.19 10^8	8.94	9.9 k_a/k_d (%)
2	2.51 10^6	3.95 10^{-1}	157	3.51 10^8	6.11	13.5 k_a/k_d (%)
3	3.84 10^6	4.31 10^{-1}	112	3.69 10^8	7.17	12 k_a/k_d (%)
Average	2.6 10^6	0.370	151	3.80 10^8		
CV (%)	36	17	19			

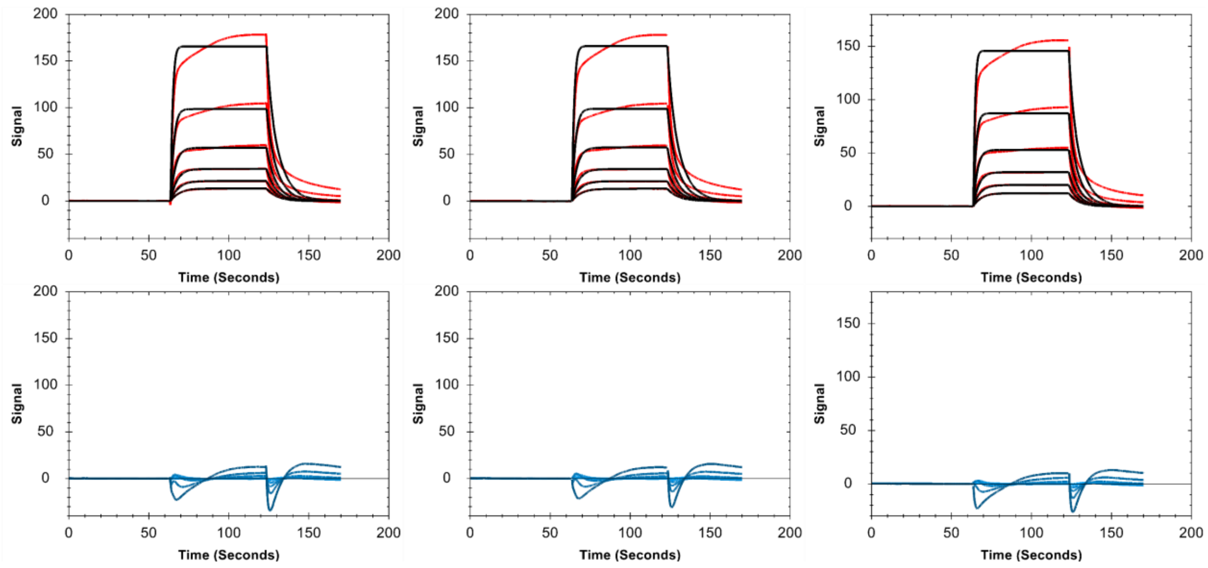

Figure S5: RRM2 binding to RNA-L1a. From left to right, replicates 1 to 3. From top to bottom, sensorgram fitted with a 1:1 model, and residuals with concentrations depicted in a blue-gradient where a darker-color implies a higher concentration.

Table S4: Kinetic and affinity values for the interaction between RRM2 and RNA-L1a corresponding to a 1:1 model fitting. Note that Chi^2 values are not corrected by the maximum signals as in the manuscript.

Replicate number	k_a (M^{-1} s^{-1})	k_d (s^{-1})	K_D (nM)	k_t (Signal/(M*s))	Chi^2 ($Signal^2$)	U-value:
1	1.70 10^6	2.23 10^{-1}	131	4.16 10^8	27	14.4 k_t (%)
2	1.49 10^6	1.99 10^{-1}	134	5.11 10^8	21.82	3.8 k_t (%)
3	2.22 10^6	2.31 10^{-1}	104	4.70 10^8	16.32	2.8 k_a/k_d (%)
Average	1.8 10^6	0.218	123	4.66 10^8		
CV (%)	17	6	11	8		

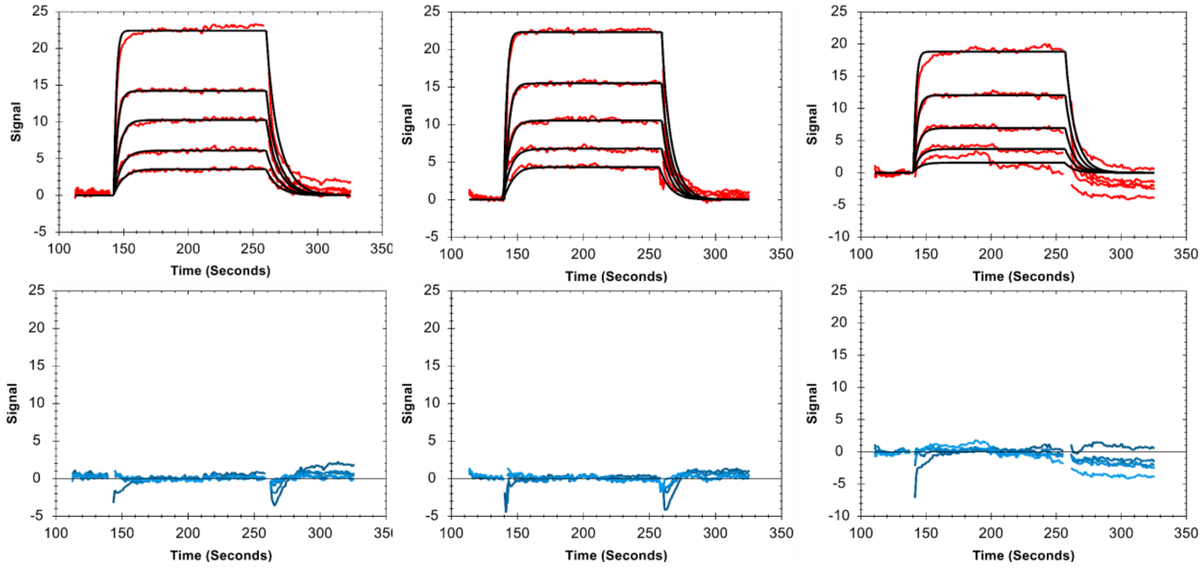

Figure S6: RRM1 binding to RNA-HP2a. From left to right, replicates 1 to 3. From top to bottom, sensorgram fitted with a 1:1 model, and residuals with concentrations depicted in a blue-gradient where a darker-color implies a higher concentration.

Table S5: Kinetic and affinity values for the interaction between RRM1 and RNA-HP2acorresponding to a 1:1 model fitting. Note that Chi^2 values are not corrected by the maximum signals as in the manuscript.

Replicate number	k_a (M^{-1} s^{-1})	k_d (s^{-1})	K_D (nM)	k_t (Signal/(M*s))	Chi^2 ($Signal^2$)	U-value:
1	9.26 10^6	1.50 10^{-1}	16.2	3.64 10^8	0.39	2.5 k_t (%)
2	7.49 10^6	2.08 10^{-1}	27.8	3.00 10^8	0.35	2.8 k_a/k_d (%)
3	8.05 10^6	1.75 10^{-1}	21.8	4.12 10^8	1.71	39.2 k_t (%)
Average	8.3 10^6	0.178	21.9	3.59 10^8		
CV (%)	9	13	22	13		

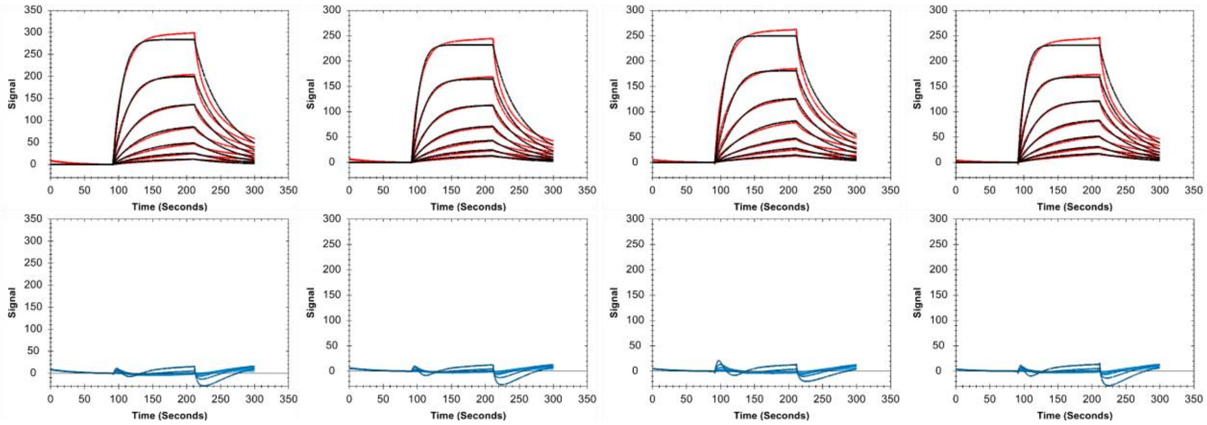

Figure S7: RRM2 binding to RNA-HP2a. From top to bottom, replicates 1 to 4. From left to right, sensorgram fitted with a 1:1 model, and residuals with concentrations depicted in a blue-gradient where a darker-color implies a higher concentration.

Table S6: Kinetic and affinity values for the interaction between RRM2 and RNA-HP2a corresponding to a 1:1 model fitting. Note that Chi^2 values are not corrected by the maximum signals as in the manuscript.

Replicate number	k_a (M^{-1} s^{-1})	k_d (s^{-1})	K_D (nM)	k_t (Signal $M^{-1}s^{-1}$)	Chi^2 ($Signal^2$)	U-value:
1	7.06 10^5	3.12 10^{-2}	44.2	2.98 10^8	36.72	3.8 k_t (%)
2	9.32 10^5	3.55 10^{-2}	3.8.1	2.72 10^8	26.58	6.1 k_t (%)
3	1.49 10^6	5.32 10^{-2}	35.7	1.43 10^8	23.69	<0.1 B_{max}/k_d (%)
4	1.07 10^6	3.27 10^{-2}	30.6	3.00 10^8	29.53	9.2 k_t (%)
Average	1.1 10^6	0.038	37.2	2.53 10^8		
CV (%)	27	23	13	26		

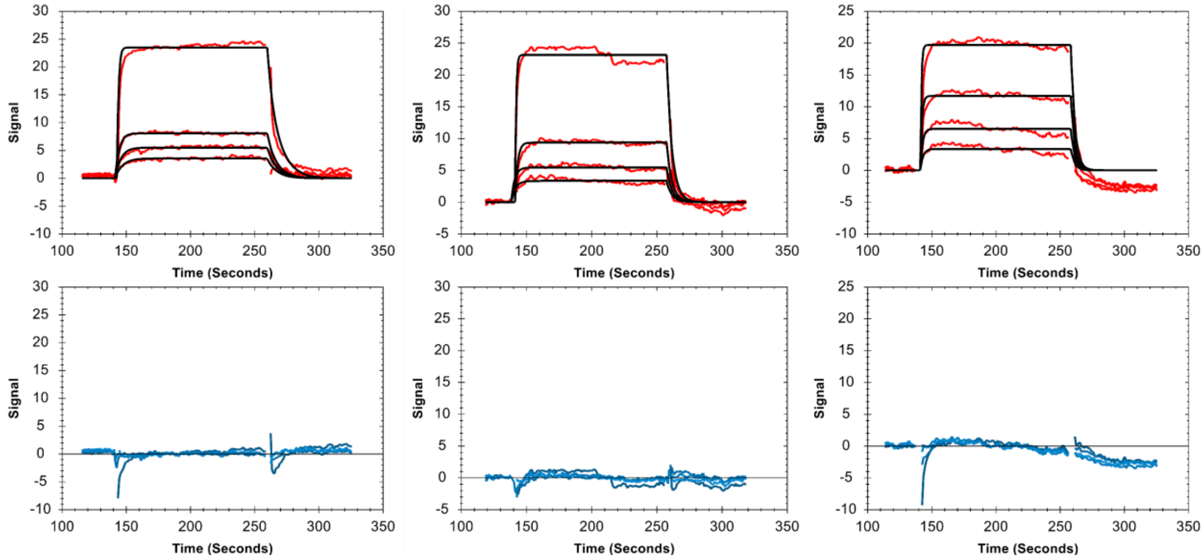

Figure S8: RRM1 binding to RNA-HP2b. From top to bottom, replicates 1 to 3. From left to right, sensorgram fitted with a 1:1 model, and residuals with concentrations depicted in a blue-gradient where a darker-color implies a higher concentration.

Table S7: Kinetic and affinity values for the interaction between RRM1 and RNA-HP2b corresponding to a 1:1 model fitting. Note that Chi^2 values are not corrected by the maximum signals as in the manuscript.

Replicate number	k_a (M^{-1} s^{-1})	k_d (s^{-1})	K_D (nM)	k_t (Signal $M^{-1}s^{-1}$)	Chi^2 (Signal^2)	U-value:
1	6.90 10^6	1.66 10^{-1}	24.1	3.07 10^8	0.55	6.2 k_t (%)
2	8.83 10^6	2.57 10^{-1}	29.0	2.86 10^9	0.48	>50 k_t (%)
3	8.44 10^6	3.82 10^{-1}	45.2	8.28 10^8	2.21	>50 k_t (%)
Average	8.0 10^6	0.268	32.8	3.59 10^8		
CV (%)	10	33	28	83		

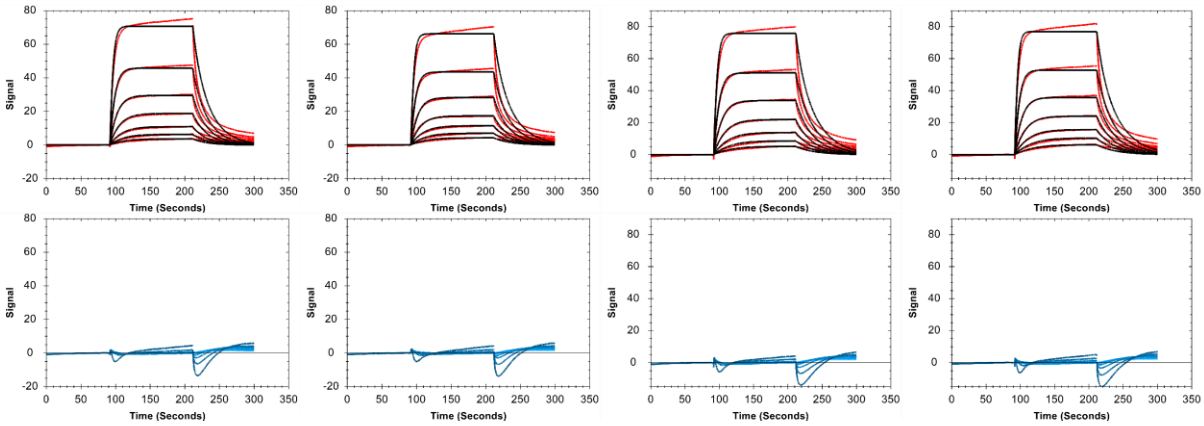

Figure S9: RRM2 binding to RNA-HP2b. From top to bottom, replicates 1 to 4. From left to right, sensorgram fitted with a 1:1 model, and residuals with concentrations depicted in a blue-gradient where a darker-color implies a higher concentration.

Table S8: Kinetic and affinity values for the interaction between RRM2 and RNA-HP2b corresponding to a 1:1 model fitting. Note that Chi^2 values are not corrected by the maximum signals as in the manuscript.

Replicate number	k_a (M^{-1} s^{-1})	k_d (s^{-1})	K_D (nM)	k_t (Signal $M^{-1}s^{-1}$)	Chi^2 (Signal^2)	U-value:
1	1.89 10^6	5.82 10^{-2}	30.7	3.75 10^8	3.85	6.5 k_t (%)
2	2.21 10^6	5.78 10^{-2}	26.2	3.02 10^8	3.97	3.6 k_d/k_t (%)
3	1.93 10^6	5.19 10^{-2}	26.9	3.00 10^8	4.78	9.8 k_t (%)
4	2.39 10^6	5.20 10^{-2}	21.8	3.33 10^8	5.47	11.4 k_t (%)
Average	2.1 10^6	0.055	26.4	3.28 10^8		
CV (%)	11	5.5	12	9		

Sensorgrams of MSI1 binding to RNA L1, L1a, HP2a and HP2b with residuals plot

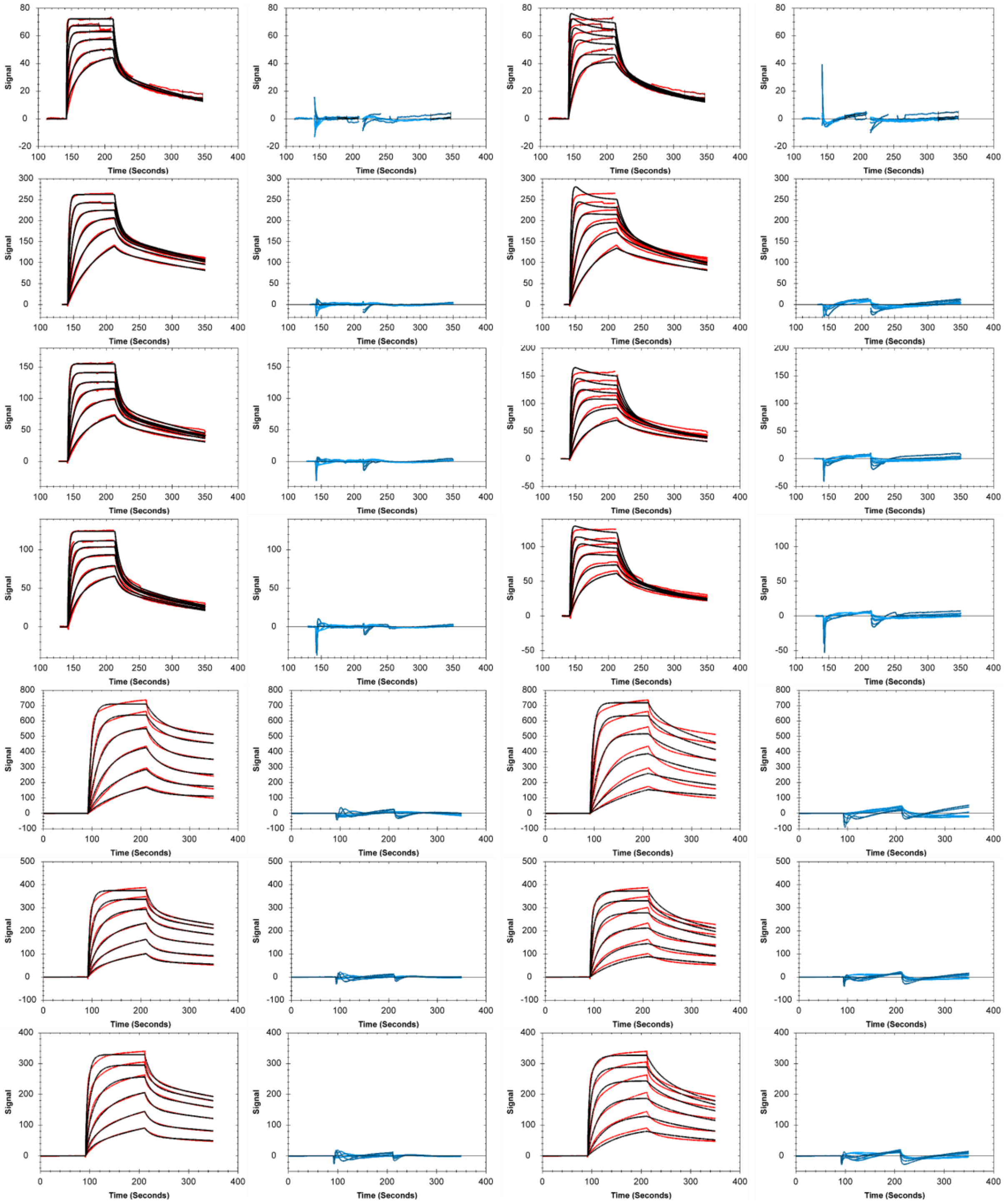

Figure S10: MSI1-RNA L1 interaction fitted with a 1:2 (left) and bivalent model (right), residuals plot corresponding to each fitting are placed right to the sensorgram in a blue gradient where a darker color implies a higher concentration. Replicate 1 to 7 are placed from top to bottom, with replicate 1 corresponding to a lower RNA coating density (39 RUs), replicates 2 to 4 to a medium RNA coating density (82 RUs) and 5 to 7 to a higher RNA coating density (245 RUs).

Table S9: Kinetic and affinity values for the interaction between MSI1 and RNA-L1 corresponding to different models. The values for the rapid 2:1 model are derived from fitting the heterogeneous ligand model and K_{D2} values are recalculated based on equation 6. Note that Chi^2 values are not corrected by the maximum signals as in the manuscript.

**HETEROGENEOUS LIGAND MODEL**								
Replicate number	k_{a1} (M^{-1} s^{-1})	k_{d1} (s^{-1})	K_{D1} (nM)	k_{a2} (M^{-1} s^{-1})	k_{d2} (s^{-1})	K_{D2} (nM)	Chi^2 (Signal^2)	U-value:
1	$5.23\ 10^{7}$	$1.47\ 10^{-1}$	2.80	$4.69\ 10^{6}$	$6.81\ 10^{-3}$	1.45	2.62	9.4 k_{a1}(%)
2	$1.95\ 10^{6}$	$1.20\ 10^{-1}$	61.3	$1.94\ 10^{6}$	$3.13\ 10^{-3}$	1.61	10.38	3.5 k_{a1}/k_{d1} (%)
3	$1.45\ 10^{6}$	$1.39\ 10^{-1}$	95.8	$2.66\ 10^{6}$	$4.89\ 10^{-3}$	1.84	5.51	3.4 k_{a1}/k_{d1} (%)
4	$1.31\ 10^{6}$	$1.25\ 10^{-1}$	95.6	$3.10\ 10^{6}$	$6.03\ 10^{-3}$	1.94	6.13	3.6 k_{a1}/k_{d1} (%)
5	$3.62\ 10^{6}$	$3.10\ 10^{-2}$	85.7	$5.55\ 10^{5}$	$3.85\ 10^{-4}$	0.69	80.61	7.8 k_{d2}(%)
6	$2.04\ 10^{6}$	$6.75\ 10^{-2}$	33.1	$6.19\ 10^{5}$	$1.44\ 10^{-3}$	2.33	19.76	4.1 k_{a1}/k_{d1} (%)
7	$2.48\ 10^{6}$	$7.17\ 10^{-2}$	28.9	$6.15\ 10^{5}$	$1.60\ 10^{-3}$	2.60	14.44	4.3 k_{a1}/k_{d1} (%)
Average	$8.9\ 10^{6}$	$1.00\ 10^{-1}$	57.6	$2.03\ 10^{6}$	$3.47\ 10^{-3}$	1.78		
CV (%)	201	40	59	72	66	32		
**BIVALENT MODEL**								
Replicate number	k_{a1} (M^{-1} s^{-1})	k_{d1} (s^{-1})	K_{D1} (nM)	k_{a2} (Signal^{-1} s^{-1})	k_{d2} (s^{-1})	K_{D2} (μM)	Chi^2 (Signal^2)	U-value:
1	$5.57\ 10^{6}$	$6.23\ 10^{-2}$	11.2	$1.45\ 10^{-3}$	$1.60\ 10^{-2}$	2.44	6.41	6.5 k_t (%)
2	$1.45\ 10^{6}$	$5.20\ 10^{-2}$	35.9	$6.42\ 10^{-4}$	$1.24\ 10^{-2}$	4.27	49.52	3.6 k_d/k_t (%)
3	$2.22\ 10^{6}$	$4.17\ 10^{-2}$	18.8	$5.60\ 10^{-4}$	$1.26\ 10^{-2}$	4.98	23.01	9.8 k_t (%)
4	$2.63\ 10^{6}$	$4.36\ 10^{-2}$	16.6	$4.89\ 10^{-4}$	$1.32\ 10^{-2}$	5.97	18.52	11.4 k_t (%)
5	$3.52\ 10^{5}$	$5.48\ 10^{-2}$	156	$1.96\ 10^{-2}$	$3.37\ 10^{-1}$	3.80	364.81	>50 k_{a2}/k_{d2} (%)
6	$7.29\ 10^{5}$	$2.04\ 10^{-2}$	28.0	$1.49\ 10^{-2}$	$3.24\ 10^{-1}$	4.81	75.64	>50 k_{a2}/k_{d2} (%)
7	$7.94\ 10^{5}$	$1.90\ 10^{-2}$	23.9	$3.08\ 10^{-2}$	$6.69\ 10^{-1}$	4.81	55.78	>50 k_{a2}/k_{d2} (%)
Average	$1.96\ 10^{6}$	$4.20\ 10^{-2}$	41.5	$9.78\ 10^{-3}$	$1.98\ 10^{-1}$	4.44	84.81	
CV (%)	84	37	114	115	120	23		
**RAPID 2:1 model**								
Replicate number	k_{a1} (M^{-1} s^{-1})	k_{d1} (s^{-1})	K_{D1} (nM)	k_{a2} (M^{-1} s^{-1})	k_{d2} (s^{-1})	K_{D2} (μM)	Chi^2 (Signal^2)	U-value:
1	$5.23\ 10^{7}$	$1.47\ 10^{-1}$	2.80	-	-	0.77	-	-
2	$1.95\ 10^{6}$	$1.20\ 10^{-1}$	61.3	-	-	1.50	-	-
3	$1.45\ 10^{6}$	$1.39\ 10^{-1}$	95.8	-	-	1.36	-	-
4	$1.31\ 10^{6}$	$1.25\ 10^{-1}$	95.6	-	-	1.98	-	-
5	$3.62\ 10^{6}$	$3.10\ 10^{-2}$	85.7	-	-	1.47	-	-
6	$2.04\ 10^{6}$	$6.75\ 10^{-2}$	33.1	-	-	1.26	-	-
7	$2.48\ 10^{6}$	$7.17\ 10^{-2}$	28.9	-	-	1.33	-	-
Average	$8.9\ 10^{6}$	$1.00\ 10^{-1}$	57.6	-	-	1.38	-	-
CV (%)	201	40	59	-	-	24	-	-

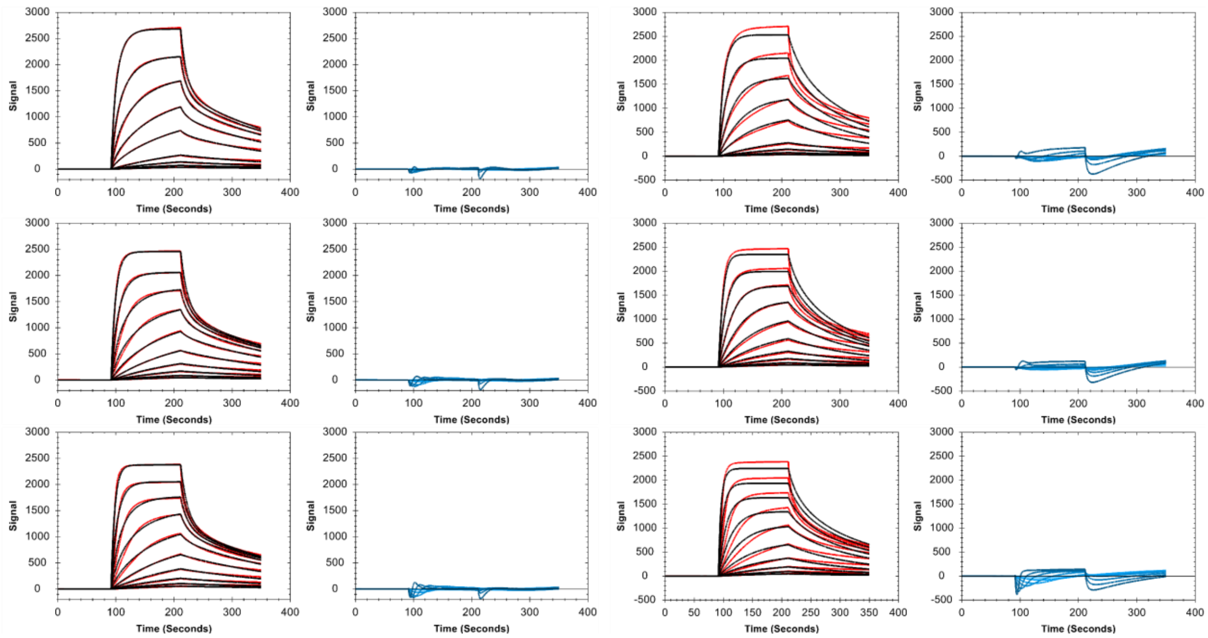

Figure S11: MSI1-RNA L1a interaction fitted with a 1:2 (left) and bivalent model (right), residuals plot corresponding to each fitting are placed right to the sensorgram in a blue gradient where a darker color implies a higher concentration.

Table S10: Kinetic and affinity values for the interaction between MSI1 and RNA-L1a corresponding to different models. Note that Chi^2 values are not corrected by the maximum signals as in the manuscript.

**HETEROGENEOUS LIGAND MODEL**								
Replicate number	k_{a1} (M^{-1} s^{-1})	k_{d1} (s^{-1})	K_{D1} (nM)	k_{a2} (M^{-1} s^{-1})	k_{d2} (s^{-1})	K_{D2} (nM)	Chi^2 (Signal^2)	U-value:
1	$6.76\ 10^4$	$9.76\ 10^{-2}$	1440	$6.61\ 10^4$	$4.74\ 10^{-3}$	71.7	190.27	2.2 k_{a2}/k_{d2} (%)
2	$7.02\ 10^4$	$8.39\ 10^{-2}$	1200	$1.06\ 10^5$	$4.81\ 10^{-3}$	45.4	375.38	3.8 k_{a1}/k_{d1} (%)
3	$9.08\ 10^4$	$8.03\ 10^{-2}$	884	$1.41\ 10^5$	$4.88\ 10^{-3}$	34.5	572.68	2.9 k_{a1}/k_{d1} (%)
Average	$7.62\ 10^4$	$8.73\ 10^{-2}$	1170	$1.04\ 10^5$	$4.81\ 10^{-3}$	50.5		
CV (%)	14	9	19	29	1	31		
**BIVALENT MODEL**								
Replicate number	k_{a1} (M^{-1} s^{-1})	k_{d1} (s^{-1})	K_{D1} (nM)	k_{a2} (Signal^{-1} s^{-1})	k_{d2} (s^{-1})	K_{D2} (µM)	Chi^2 (Signal^2)	U-value:
1	$6.27\ 10^4$	$3.61\ 10^{-2}$	576	2.1910^{-3}	1.53	155	4165.23	>50 k_{a2}/k_{d2} (%)
2	$5.24\ 10^4$	$1.69\ 10^{-1}$	3230	$3.11\ 10^{-2}$	5.63	40.1	2826.91	>50 k_{a2}/k_{d2} (%)
3	$1.37\ 10^4$	$5.79\ 10^{-2}$	422	$1.79\ 10^{-3}$	$5.25\ 10^{-1}$	64.9	4857.39	>50 k_{a2}/k_{d2} (%)
Average	$8.40\ 10^4$	$8.77\ 10^{-2}$	1410	$1.17\ 10^{-2}$	2.56	86.5		
CV (%)	45	66	91	117	86	57		
**RAPID 2:1 model**								
Replicate number	k_{a1} (M^{-1} s^{-1})	k_{d1} (s^{-1})	K_{D1} (nM)	k_{a2} (M^{-1} s^{-1})	k_{d2} (s^{-1})	K_{D2} (µM)	Chi^2 (Signal^2)	U-value:
1	$6.76\ 10^4$	$9.76\ 10^{-2}$	1440	-	-	63.1	-	-
2	$7.02\ 10^4$	$8.39\ 10^{-2}$	1200	-	-	59.9	-	-
7	$9.08\ 10^4$	$8.03\ 10^{-2}$	884	-	-	57.8	-	-
Average	$7.62\ 10^4$	$8.73\ 10^{-2}$	1170	-	-	60.3	-	-
CV (%)	14	9	19	-	-	4	-	-

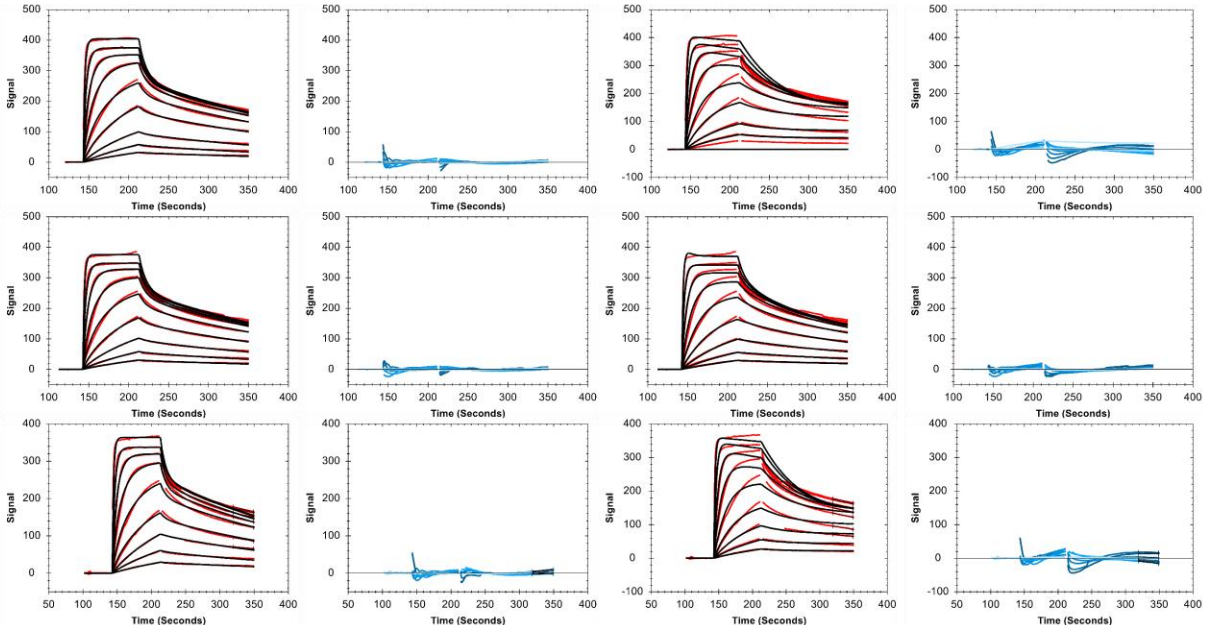

Figure S12: MSI1-RNA HP2a interaction fitted with a 1:2 (left) and bivalent model (right), residuals plot corresponding to each fitting are placed right to the sensorgram in a blue gradient where a darker color implies a higher concentration.

Table S11: Kinetic and affinity values for the interaction between MSI1 and RNA-HP2a corresponding to different models. Note that Chi^2 values are not corrected by the maximum signals as in the manuscript.

**HETEROGENEOUS LIGAND MODEL**								
Replicate number	k_{a1} (M^{-1} s^{-1})	k_{d1} (s^{-1})	K_{D1} (nM)	k_{a2} (M^{-1} s^{-1})	k_{d2} (s^{-1})	K_{D2} (nM)	Chi^2 (Signal^2)	U-value:
1	$1.78\ 10^{6}$	$1.11\ 10^{-1}$	62.4	$8.65\ 10^{5}$	$3.65\ 10^{-3}$	4.22	18.07	4.1 k_{a2}/k_{d2} (%)
2	$1.35\ 10^{6}$	$1.15\ 10^{-1}$	84.6	$9.24\ 10^{5}$	$3.81\ 10^{-3}$	4.12	15.59	4.4 k_{a2}/k_{d2} (%)
3	$1.56\ 10^{6}$	$1.30\ 10^{-1}$	83.3	$9.38\ 10^{5}$	$3.72\ 10^{-3}$	3.97	18	5.5 k_{a2}/k_{d2} (%)
Average	$1.56\ 10^{6}$	$1.19\ 10^{-1}$	76.8	$9.09\ 10^{5}$	$3.73\ 10^{-3}$	4.10		
CV (%)	11	7	13	3	2	3		
**BIVALENT MODEL**								
Replicate number	k_{a1} (M^{-1} s^{-1})	k_{d1} (s^{-1})	K_{D1} (nM)	k_{a2} (Signal^{-1} s^{-1})	k_{d2} (s^{-1})	K_{D2} (µM)	Chi^2 (Signal^2)	U-value:
1	$9.70\ 10^{5}$	$1.28\ 10^{-2}$	13.2	$7.46\ 10^{-5}$	$1.02\ 10^{-10}$	$3.02\ 10^{-7}$	157.02	>50 k_{d2} (%)
2	$6.54\ 10^{5}$	$7.23\ 10^{-2}$	110	$1.25\ 10^{-3}$	$2.66\ 10^{-2}$	4.71	35.07	12 k_{a2}/k_{d2} (%)
3	$1.07\ 10^{6}$	$1.14\ 10^{-2}$	10.6	$8.28\ 10^{-5}$	$5.79\ 10^{-12}$	$1.54\ 10^{-8}$	96.49	>50 k_{d2} (%)
Average	$8.98\ 10^{5}$	$3.22\ 10^{-2}$	44.6	$4.69\ 10^{-4}$	$8.87\ 10^{-3}$	1.57		
CV (%)	20	88	104	118	141	141		
**RAPID 2:1 model**								
Replicate number	k_{a1} (M^{-1} s^{-1})	k_{d1} (s^{-1})	K_{D1} (nM)	k_{a2} (M^{-1} s^{-1})	k_{d2} (s^{-1})	K_{D2} (µM)	Chi^2 (Signal^2)	U-value:
1	$1.78\ 10^{6}$	$1.11\ 10^{-1}$	62.4	-	-	3.36	-	-
2	$1.35\ 10^{6}$	$1.15\ 10^{-1}$	84.6	-	-	3.26	-	-
7	$1.56\ 10^{6}$	$1.30\ 10^{-1}$	83.3	-	-	2.67	-	-
Average	$1.56\ 10^{6}$	$1.19\ 10^{-1}$	76.8	-	-	3.09	-	-
CV (%)	11	7	13	-	-	10	-	-

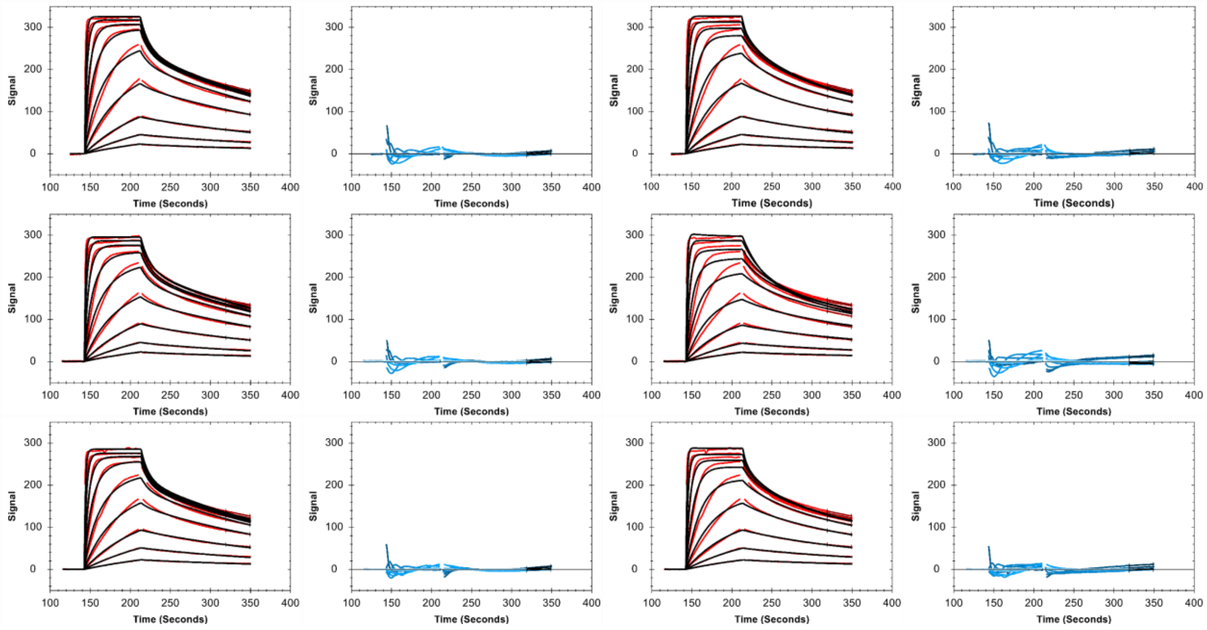

Figure S13: MSI1-RNA HP2b interaction fitted with a 1:2 (left) and bivalent model (right), residuals plot corresponding to each fitting are placed right to the sensorgram in a blue gradient where a darker color implies a higher concentration.

Table S12: Kinetic and affinity values for the interaction between MSI1 and RNA-HP2b corresponding to different models. Note that Chi^2 values are not corrected by the maximum signals as in the manuscript.

**HETEROGENEOUS LIGAND MODEL**								
Replicate number	k_{a1} (M^{-1} s^{-1})	k_{d1} (s^{-1})	K_{D1} (nM)	k_{a2} (M^{-1} s^{-1})	k_{d2} (s^{-1})	K_{D2} (nM)	Chi^2 (Signal^2)	U-value:
1	2.14 10^6	8.84 10^{-2}	41.3	1.06 10^6	3.91 10^{-3}	3.69	27.72	6.5 k_{a2}/k_{d2} (%)
2	1.96 10^6	8.31 10^{-2}	42.3	1.18 10^6	3.94 10^{-3}	3.35	21.61	6.1 k_{a2}/k_{d2} (%)
3	1.73 10^6	1.08 10^{-1}	62.3	1.20 10^6	4.30 10^{-3}	3.57	16.7	6 k_{a2}/k_{d2} (%)
Average	1.94 10^6	9.32 10^{-2}	48.6	1.15 10^6	4.05 10^{-3}	3.54		
CV (%)	9	11	20	5	4	4		
**BIVALENT MODEL**								
Replicate number	k_{a1} (M^{-1} s^{-1})	k_{d1} (s^{-1})	K_{D1} (nM)	k_{a2} (Signal^{-1} s^{-1})	k_{d2} (s^{-1})	K_{D2} (µM)	Chi^2 (Signal^2)	U-value:
1	6.37 10^5	1.59 10^{-1}	250	6.51 10^{-3}	5.63 10^{-2}	1.91	39.18	18 k_{d2} (%)
2	1.09 10^6	1.91 10^{-2}	17.6	6.35 10^{-4}	2.37 10^{-2}	8.26	56.76	28.1 k_{a2}/k_{d2} (%)
3	7.53 10^5	1.23 10^{-1}	164	3.85 10^{-3}	4.09 10^{-2}	2.35	27.26	12.8 k_{a2}/k_{d2} (%)
Average	8.27 10^5	1.00 10^{-1}	144	3.67 10^{-3}	4.03 10^{-2}	4.17		
CV (%)	23	59	67	66	33	69		
**RAPID 2:1 model**								
Replicate number	k_{a1} (M^{-1} s^{-1})	k_{d1} (s^{-1})	K_{D1} (nM)	k_{a2} (M^{-1} s^{-1})	k_{d2} (s^{-1})	K_{D2} (µM)	Chi^2 (Signal^2)	U-value:
1	2.14 10^6	8.84 10^{-2}	41.3	-	-	3.37	-	-
2	1.96 10^6	8.31 10^{-2}	42.3	-	-	2.92	-	-
7	1.73 10^6	1.08 10^{-1}	62.3	-	-	2.81	-	-
Average	1.94 10^6	9.32 10^{-2}	48.6	-	-	3.03	-	-
CV (%)	9	11	20	-	-	8	-	-

InteractionMap analysis of MSI1 binding to RNA HP2a and HP2b

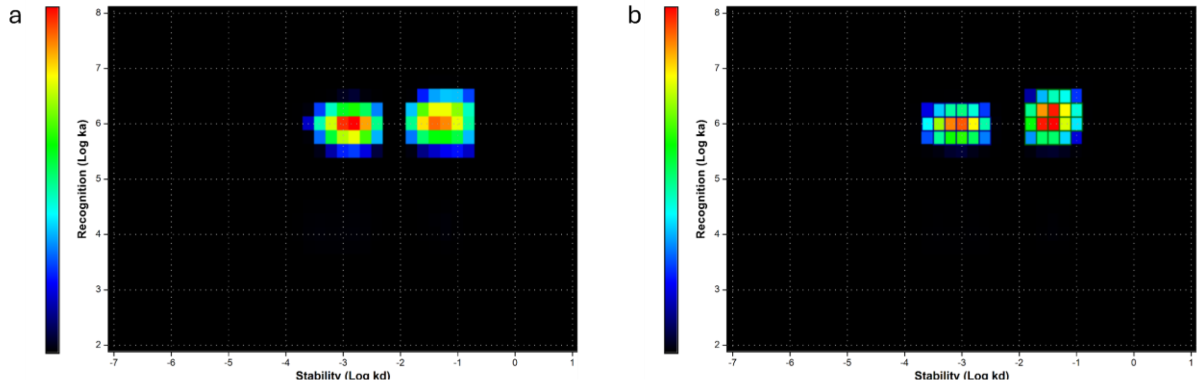

Figure S14: a) InteractionMap analysis corresponding to a MSI1-RNA HP2a interaction. b) InteractionMap corresponding to a MSI1-RNA HP2b interaction.

Sensorgrams of MSI1 binding to HP2a and HP2b with residuals plot fitted with an induced fit model.

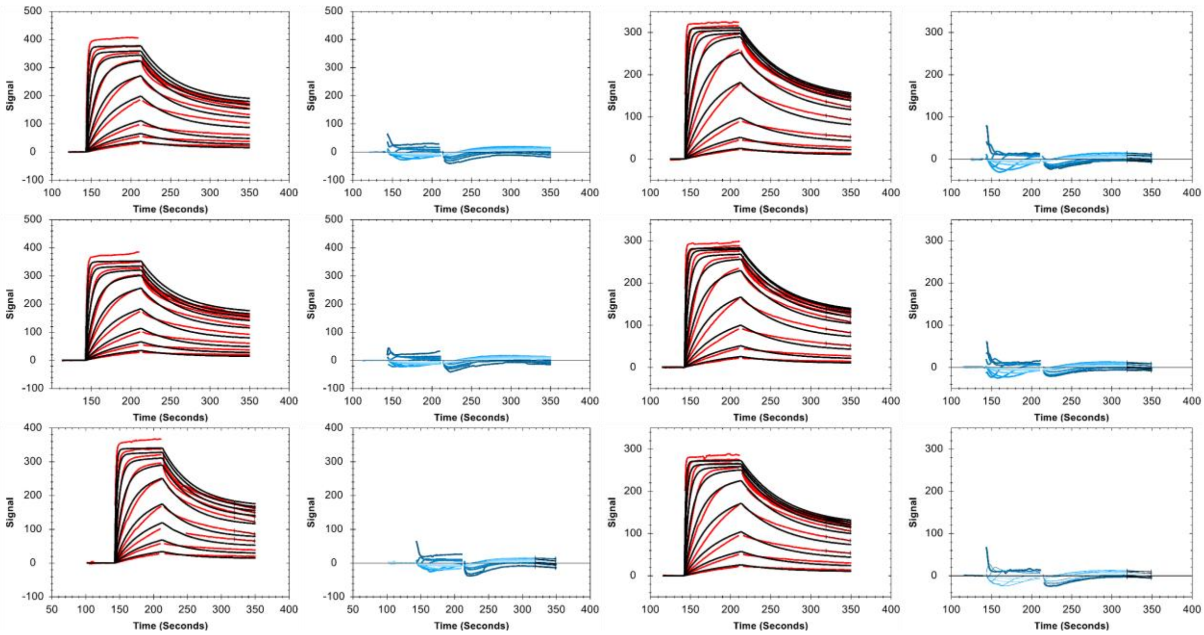

Figure S15: On the left side, MSI1-RNA HP2a interaction fitted with an induced fit model. On the right side, MSI1-RNA HP2b interaction fitted with an induced fit model. Residuals plot corresponding to each fitting are placed right to the sensorgram in a blue gradient where a darker color implies a higher concentration.

Table S13: Kinetic and affinity values for the interaction between MSI1 and RNA-HP2 and RNA-HP2b corresponding to an induced fit model fitting. Note that Chi^2 values are not corrected by the maximum signals as in the manuscript.

**INDUCED FIT MODEL – RNA HP2a**							
Replicate number	k_{a1} (M^{-1} s^{-1})	k_{d1} (s^{-1})	k_{a2} (s^{-1})	k_{d2} (s^{-1})	K_{D2} (M)	Chi^2 ($Signal^2$)	U-value:
1	$1.06\ 10^{6}$	$1.69\ 10^{-2}$	$5.56\ 10^{-3}$	$1.53\ 10^{-8}$	$4.37\ 10^{-14}$	168.15	>50 k_{d2} (%)
2	$1.07\ 10^{6}$	1.6910^{-2}	$5.51\ 10^{-3}$	$1.42\ 10^{-4}$	$4.05\ 10^{-10}$	129.89	>50 k_{d2} (%)
3	$1.12\ 10^{6}$	$1.65\ 10^{-2}$	$5.60\ 10^{-3}$	$2.24\ 10^{-8}$	$5.86\ 10^{-14}$	124.05	>50 k_{d2} (%)
Average	$1.08\ 10^{6}$	$1.68\ 10^{-2}$	$5.56\ 10^{-3}$	$4.73\ 10^{-5}$	$1.35\ 10^{-10}$		
CV (%)	2	1	1	141	141		
**INDUCED FIT MODEL – RNA HP2b**							
Replicate number	k_{a1} (M^{-1} s^{-1})	k_{d1} (s^{-1})	k_{a2} (s^{-1})	k_{d2} (s^{-1})	K_{D2} (M)	Chi^2 ($Signal^2$)	U-value:
1	$1.22\ 10^{6}$	$1.29\ 10^{-2}$	$4.53\ 10^{-3}$	$1.78\ 10^{-10}$	$4.15\ 10^{-16}$	100.71	>50 k_{d2} (%)
2	$1.32\ 10^{6}$	$1.39\ 10^{-2}$	$4.58\ 10^{-3}$	$5.58\ 10^{-11}$	$1.28\ 10^{-16}$	75.33	>50 k_{d2} (%)
3	$1.34\ 10^{6}$	$1.39\ 10^{-2}$	$4.34\ 10^{-3}$	$1.61\ 10^{-10}$	$3.84\ 10^{-16}$	81.47	>50 k_{d2} (%)
Average	$1.29\ 10^{6}$	$1.36\ 10^{-2}$	$4.48\ 10^{-3}$	$5.40\ 10^{-11}$	$3.09\ 10^{-16}$		
CV (%)	4	3	1	41	42		
